# Cross-Cultural Bayesian Network Analysis of Factors Affecting Residents’ Concerns About the Spread of an Infectious Disease Caused by Tourism

**DOI:** 10.3389/fpsyg.2021.635110

**Published:** 2021-06-07

**Authors:** Fumiko Kano Glückstad, Uffe Kock Wiil, Marjan Mansourvar, Pernille Tanggaard Andersen

**Affiliations:** ^1^Department of Management, Society and Communication, Copenhagen Business School, Frederiksberg, Denmark; ^2^Center of Health Informatics and Technology, Maersk Mc-Kinney Moller Institute, University of Southern Denmark, Odense, Denmark; ^3^Department of Public Health, University of Southern Denmark, Esbjerg, Denmark

**Keywords:** COVID-19, Probabilistic Structural Equation Modeling, Bayesian network, health risk perception, human values, experience economy, international tourism, cultural sensitivity

## Abstract

COVID-19 has had a severe impact globally, and the recovery can be characterized as a tug of war between fast economic recovery and firm control of further virus-spread. To be prepared for future pandemics, public health policy makers should put effort into fully understanding any complex psychological tensions that inherently arise between opposing human factors such as free enjoyment versus self-restriction. As the COVID-19 crisis is an unusual and complex problem, combinations of diverse factors such as health risk perception, knowledge, norms and beliefs, attitudes and behaviors are closely associated with individuals’ intention to enjoy the experience economy but also their concerns that the experience economy will trigger further spread of the infectious diseases. Our aim is to try identifying what factors are associated with their concerns about the spread of the infectious disease caused by the local experience economy. Hence, we have chosen a “data-driven” explanatory approach, “Probabilistic Structural Equational Modeling,” based on the principle of Bayesian networks to analyze data collected from the following four countries with indicated sample sizes: Denmark (1,005), Italy (1,005), China (1,013), and Japan (1,091). Our findings highlight the importance of understanding the contextual differences in relations between the target variable and factors such as personal value priority and knowledge. These factors affect the target variable differently depending on the local severity-level of the infections. Relations between pleasure-seeking via the experience economy and individuals’ anxiety-level about an infectious hotspot seem to differ between East Asians and Europeans who are known to prioritize so-called interpersonal- and independent self-schemes, respectively. Our study also indicates the heterogeneity in the populations, i.e., these relations differ within the respective populations. Another finding shows that the Japanese population is particularly concerned about their local community potentially becoming an infectious hotspot and hence expecting others to comply with their particular social norms. Summarizing, the obtained insights imply the importance of considering both cultural- and individual contexts when policy makers are going to develop measures to address pandemic dilemmas such as maintaining public health awareness and accelerating the recovery of the local experience economy.

## Introduction

The World Health Organization (WHO) has defined the COVID-19 pandemic as a Public Health Emergency of International Concern (PHEIC) on January 30, 2020. Since then, the Experience Economy–including international tourism–has been severely hit and it has been challenging to maintain workplaces for employees involved in this sector (Gössling et al., 2020). On the other hand, it is also evident that this global health-crisis has been inherently accelerated by people’s traveling activities within and across national borders (Chinazzi et al., 2020). Hence, this global crisis can be characterized as a tag of war between obtaining economic recovery and maintaining a firm control over further virus-spread. To be prepared for future pandemics, public health policy makers should put effort into fully understanding any complex psychological tensions that inherently arise between opposing human factors such as free enjoyment versus self-restriction. To tackle this challenge, “bottom-up individual and household measures are crucial for prevention and emergency response of the COVID-19 pandemic” (Chan et al., 2020, p1).

Individuals’ responses to the COVID-19 are associated with various factors. One of the important factors is risk perception. Risk perceptions are beliefs about potential harm or the possibility of a loss. It is a subjective judgment that people make about the characteristics and severity of a risk. Many studies have been conducted at the early stage of the current pandemic crisis in various cultural contexts. Faasse and Newby (2020), for instance, investigated relations between cognitive (e.g., perceived risk and knowledge) and affective (e.g., concerns and uncertainty) factors and health-protective behaviors among Australians. Their results identified that the level of engagement in the health protective behaviors are closely connected with psychological and demographic factors. Simione and Gnagnarella (2020) investigated relations between demographic and psychological factors and risk perception among health workers and the general population in Italy. Their findings indicated that people living in a high-risk residence area or people having a high-risk occupation increased their perceived stress and anxiety. Shiina et al. (2020) analyzed how factors such as age, education, and anxiety about the COVID-19, access to information about the COVID-19 and health-protective behaviors affect the level of knowledge about the COVID-19 among the Japanese population. An important lesson from this study was that the level of knowledge about COVID-19 correlated with their anxiety about their health status and their health protective behaviors. Wang et al. (2020)’s study reported an association of risk communication, risk perception and behavioral adherence during the COVID-19 pandemic. Moreover, some studies addressed factors influencing rather specific behaviors such as mask wearing in Japan (Nakayachi et al., 2020), social distancing in China (Xie et al., 2020), hygiene-related and avoidance-related behaviors in Australia (Seale et al., 2020), and consumers’ stockpiling behaviors during the COVID-19 crisis in Denmark (Dammeyer, 2020).

Whereas some of the associations between cognitive and affective factors and health-protective attitudes and behaviors to the COVID-19 may commonly be observed in different cultural contexts, some cause-effect relations may depend not only on a cultural context but also on a personal context. Muto et al. (2020) addressed Japanese populations’ behavioral changes at the early stage of the crisis during January–March 2020, which depended on *individuals’ self-restraint*. The study by Muto et al. (2020) demonstrated that the majority of the population over 40 years old followed various recommendations on health-protective behaviors, and they trusted information from the central and local governments. However, their study also indicated that a younger and unmarried segment with a drinking and smoking habit coming from lower-income households had a reluctant tendency to accord with the recommendations issued by the authorities. Lower engagement in implementing the measures on the COVID-19 were also observed among specific personality traits, among others risk taking traits (Howard, 2021), antisocial risk takers (Zajenkowski et al., 2020), anti-social traits (Miguel et al., 2020), and dark triad trait (Nowak et al., 2020) in various cultural contexts.

Although the cognitive-, affective-, psychographic-, and sociodemographic factors associating with their health-related concerns and their health protective behaviors have generally been investigated in previous research, there are a limited number of studies investigating individuals’ traveling behaviors and their health-related concerns and protective behaviors. Shamsrizi et al. (2020) reported, from the viewpoint of travel medicine practitioners, that “the vast majority of travelers visiting (their center) did not appreciate the health risks and logistical challenges posed by the evolving pandemic just before the international ban on travel and the near to complete lockdown on international air travel” (Shamsrizi et al., 2020, p1640). Their study pointed out the heterogeneous “response to an imminent epidemiological threat” (Shamsrizi et al., 2020, p1640) observed among the German population. Similarly, a qualitative study by Ma et al. (2020) on risk perception of Chinese international students traveling to Australia indicated that those Chinese international students “could be a risk population for importations of infections such as COVID-19 because of low risk perception and lack of seeking travel health advice” (Ma et al., 2020, p197). On the contrary, the study by Parady et al. (2020) investigated travel behavior changes during the COVID-19 pandemic in Japan and reported that not only the risk perception, but also “*the perception of self-restriction of others*” had effects on the moderate reduction in shopping, eating-out and leisure activities.

The previous works highlight that factors affect individuals’ concerns about COVID-19 and their protective behaviors against it are diverse. Furthermore, individuals’ health risk perception, knowledge, norms and beliefs, attitudes and behaviors (self-protective and/or socially responsible) are closely associated with their intention to enjoy the experience economy but also their concerns that the experience economy will trigger further spread of the infectious diseases. From the public health perspective, scientific evidence addressing this complex psychological tension between the enjoyment and self-restriction is highly relevant and important. For policy makers, understanding such problem must provide insights about why a specific measure or a policy may work for some countries, but not for others. Finally, from the view of the experience economy industry, it is also important to understand the dilemma that the industry needs to maintain workplaces for those working in this sector, while maintaining the safety of local residents. As the tourism researchers tend to have stronger attention to the recovery of the experience economy in the post-corona era, there is an insufficient number of studies that cross-culturally investigate scientific evidence of this complex psychological tension between the enjoyment and the self-restriction related to this crisis. Accordingly, this article attempts to dive into the complex entanglements, i.e., what factors are associated with individuals’ concerns about the spread of the infectious disease caused by the local experience economy generating an influx of international foreign tourism. Our assumption is that cultural norms and one’s societal environment may be a moderator of the complex relations between various factors and this target variable. Hence, this article will highlight cultural differences in various factors affecting this specific target variable. We will analyze data collected from four selected countries, Denmark, Italy, Japan, and China. Denmark is highlighted as one of the countries that handled the pandemic in a timely manner in the first phase (Oksanen et al., 2020; Olagnier and Mogensen, 2020). Italy is the first country in Europe that was declared as the European Epicenter at the first phase of the pandemic. China as the first country hit by the pandemic. They have lived longer with the pandemic. Finally, Japan succeeded in controlling COVID-19 by depending on *individuals’ self-restraint* in the first phase. However, this situation has changed since then and been criticized regarding their lack of testing capacity (Shimizu et al., 2020). At the time of the first phase, Japan was supposed to host the 2020 Tokyo Olympic Games originally planned in July–August 2020. Subsequently, we will interpret the results of our explanatory data analysis considering the various cultural and personal contexts.

## Materials and Methods

### Research Strategy

Whereas the hypothesis quantitative research is classified as the positivist epistemology (Karasz and Singelis, 2009), we found that there may be a limitation to rely on the positivistic approach where numerous theories could be applied or not at all applied to explain the current complex and unusual situations of the COVID-19 crisis observed in various cultural contexts. Accordingly, we have chosen a pragmatic approach to understand the reality of the COVID-19 crisis. Specifically, we have selected numerous questions based on our review of the existing COVID-19 related literature and assumed that several theoretical constructs (e.g., risk perception, social responsibility) may be extracted through explorative data analysis.

In the field of cross-cultural psychology, the conventional data analysis methods having been typically used are the multi-group regression or multi-group structural equational modeling, which is the explanatory modeling focusing on the function *f* in the formula: *Y* = *f*(*X*). In the positivist epistemology, the formula of *f*(*X*) is defined by a researcher who establish a theory in the form of a path model. Instead, our study attempted to discover potential theoretical constructs *f*(*X*) that explain about a specific target phenomenon *Y* without defining a specific path model. For this purpose, our study has chosen a data-driven explanatory modeling called Probabilistic Structural Equation Modeling (PSEM) available in the BayesiaLab software (Conrady and Joufee, 2015) used in various scientific fields (e.g., Seixas et al., 2017; Seixas et al., 2018; Gerassis et al., 2019). The BayesiaLab software is based on the principle of Bayesian networks (Pearl, 2009). A Bayesian network is a representation of systems where nodes displaying the variables of interest are linked as a form of network. The benefit of Bayesian networks is that all cause-effect assumptions, “from primary cause to final outcome” (Chen and Pollino, 2012, p134) are explicitly visualized by the use of the conditional probability tables attached to each node (variable) in the network (Heckerman, 1997; Chen and Pollino, 2012; Conrady and Joufee, 2015). Once a Bayesian network is fully developed, the joint probability distribution of the network “can be used for computing the posterior probabilities of any subset of variables given evidence about any other subset” (Conrady and Joufee, 2015, p23). Our study further exploited the simulation and optimization functions of PSEM which utilize the conditional probability tables attached to the respective variables. The optimization function enabled us to simulate what combination (chain) of factors most likely maximizes or minimizes a response to the target variable.

### Measures

Several question items that may extract potential theoretical constructs were selected. The constructs that were assumed in accordance with our literature review are listed in Table 1 that summarizes survey questions and response categories analyzed in this study. The survey questions were translated into the respective local languages and reviewed by at least two or more native speakers in the respective countries.

**TABLE 1:** Survey questions and response categories.

**Survey questions**	**Response categories**
Personal values: • He/She avoids anything that might endanger his/her safety (Security: SC1) • Excitement in life is important to him/her (Stimulation: ST1) • Being very successful is important to him/her (Achievement: AC1) • He/She strongly believes that he/she should care for nature (Universalism: UN1) • Having a good time is important to him/her (Hedonism: HD1) • It is important to him/her to maintain traditional values or beliefs (Tradition: TR1) • Being creative is important to him/her (Self-Direction: SD1) • Having the feeling of power that money can bring is important to him/her (Power: PO1) • It is important to him/her to avoid upsetting other people (Conformity: CO1) • Caring for the well-being of people he/she is close to is important to him/her (Benevolence: BE1) • He/She believes he/she should always do what people in authority say (CO2) • He/She takes advantage of every opportunity to have fun (HD2) • It is important to him/her to be loyal to those who are close to him/her (BE2) • It is important to him/her to be humble (TR2) • It is important to him/her to listen to people who are different from him/her (UN2) • It is important to him/her to make his/her own decision about his/her life (SD2) • He/She is always looking for different kinds of things to do (ST2) • He/She wants people to admire his achievements (AC2) • He/She things it is important that every person in the world has equal opportunity in life (UN3) • It is important to him/her that his/her country protects itself against all threats (SC2) • He/She wants people to do what he/she says (PO2)	1. Not at all like me 2. Not like me 3. A little like me 4. Somewhat like me 5. Like me 6. Very much like me

Travel experience: • How many times have you traveled overseas for business purposes, within the last 2 years? • How many times have you traveled overseas for leisure purposes, within the last 2 years? • How many times have you traveled domestically for leisure purposes (with an overnight stay), within the last 2 years?	1. Not at all 2. 1–3 times 3. 4–6 times 4. 7–12 times 5. 13+ times

COVID-19 experience: • Do/Did you have an infection with the COVID-19 virus? Please select one of the following options.	1. Yes, I had COVID-19, confirmed by a lab test 2. Yes, a health care provider told me that I might had/have it, but a lab test did not confirm it 3. I think I had or currently have COVID-19, but a health care provider did not confirm it 4. No, I do not think I had or currently have it 5. A test confirmed that I do/did not have it

COVID-19 knowledge: • The virus survives for days outside the body in the open air • Most people who get COVID-19 get very ill • Only elderly people die from COVID-19 • Wearing masks will prevent being infected • Smokers who get COVID-19 are more likely to get severely ill than non-smokers • You can have the virus without any symptoms • On average, children get less ill from the virus than adults	1. True 2. False

COVID-19 risk perception: • I am worried that I will become infected with COVID-19 • I am worried that I will become seriously ill after being infected with COVID-19 • I am worried that I will infect my family member if I become infected with COVID-19	1. Strongly disagree 2. Disagree 3. Somewhat disagree 4. Neither disagree nor agree 5. Somewhat agree 6. Agree 7. Strongly agree

Risk avoidance of COVID-19 infections • I avoid using public transportation to reduce the risk of being infected by the Corona-virus • I avoid larger groups in order to avoid the risk of being infected by the Corona-virus • I will choose less crowded destination in my next trip instead of visiting popular and crowded places • I will chose my next travel destination where hygiene in the public space is well maintained • I will not travel to a country with high reproduction number of infections in the near future	1. Strongly disagree 2. Disagree 3. Somewhat disagree 4. Neither disagree nor agree 5. Somewhat agree 6. Agree 7. Strongly agree

Intention to enjoy experience economy • I enjoy cafes, restaurants, shops and entertainments, as soon as the society has re-opened (EE) • I will travel abroad as soon as the borders are re-opened (travel)	1. Strongly disagree 2. Disagree 3. Somewhat disagree
	4. Neither disagree nor agree 5. Somewhat agree 6. Agree 7. Strongly agree

Attitudes to local businesses • It is important for our local businesses to have foreign tourists visiting to our local community • Tourists visiting our local community should behave properly in order to avoid potential risk of spreading infectious diseases • The authorities should restrict international tourism in order to avoid risk of spreading infectious diseases in our community for the next 18 months • Our local businesses should contribute to make our community clean and safe so that foreign tourists will feel safe and comfortable	1. Strongly disagree 2. Disagree 3. Somewhat disagree 4. Neither disagree nor agree 5. Somewhat agree 6. Agree 7. Strongly agree

Attitudes to responsible behaviors • I clean up a public space (e.g., toilet) after I use it so that people who use it after me feel clean and safe • I carry and use disinfectant to clean my hand before touching items so that other people who touch after me feel clean and safe • I wear a mask to keep those around me safe and comfortable	1. Strongly disagree 2. Disagree 3. Somewhat disagree 4. Neither disagree nor agree 5. Somewhat agree 6. Agree 7. Strongly agree

Attitudes to self-protective behaviors • I carry and use disinfectant to clean my hand after touching items in shops to make me feel clean and safe • I wear a mask to make me feel safe • I am keeping social distances in public spaces. If it is not possible, I will leave the place	1. Strongly disagree 2. Disagree 3. Somewhat disagree 4. Neither disagree nor agree 5. Somewhat agree 6. Agree 7. Strongly agree

Attitudes to public behaving responsible: • It is important that individuals contribute to minimize the risk of spreading infectious diseases in public spaces • I feel safe and comfortable if staffs in hotels, airlines, restaurants etc. wear a mask	1. Strongly disagree 2. Disagree 3. Somewhat disagree 4. Neither disagree nor agree 5. Somewhat agree 6. Agree 7. Strongly agree

Concern about the hot-spot • It is concerning that our community will be crowded by foreign tourists and will potentially become a hot-spot of infectious diseases	1. Strongly disagree 2. Disagree 3. Somewhat disagree 4. Neither disagree nor agree 5. Somewhat agree 6. Agree 7. Strongly agree

Gender	1. Male 2. Female

Age	1. 18–24 years old 2. 25–34 years old 3. 35–44 years old 4. 45–54 years old 5. 55+ years old
	

#### Target Variable

The COVID-19 pandemic can be characterized as a tag of war between obtaining economic recovery and maintaining a firm control over further virus-spread. This macro-economic phenomenon could be seen as the reflection of individuals’ complex psychological tension between the enjoyment and self-restriction. One way to investigate this tension is to define a target variable addressing one of these variables, such as “intention to travel abroad” referring to enjoyment. However, such behavioral intention could be influenced by a wider range of sociodemographic factors such as income and previous travel experiences. Instead of addressing individuals’ intention to enjoy traveling, our study focused on the view of residents in a local community in the perspective of sustainable tourism. Our assumption was that the local residents may express either of the opposing attitudes in the post-corona era: one supporting the local businesses involved in the experience economy and one having anxiety that the experience economy (in particular, international tourism) will attract crowds of visitors which eventually creates a hotspot of an infectious disease. Accordingly, an instruction was given as follows: “please consider the next statement from the view of resident and tell us to which extent you agree or disagree with the following statement.” The statement was described as: “It is concerning that our community will be crowded by foreign tourists and will potentially become a hotspot of infectious diseases (referred to as *individuals’ anxiety about a hotspot*).”

#### Personal Values

**FIGURE 1:**
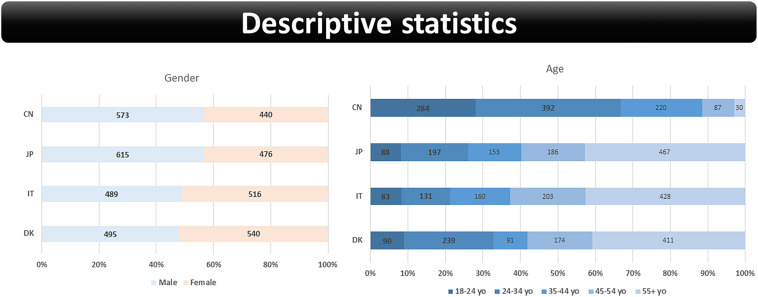
Gender and age of the respondents.

Individuals’ personality traits are important factors affecting their response to a crisis, such as the COVID-19 pandemic. Wolf et al. (2020) argue that human values play a critical role “in driving both behavioral compliance to government guidelines and promoting prosocial behaviors to alleviate the strains arising from a prolonged pandemic” (Wolf et al., 2020, p618). The human values also explain motivational drivers of enjoyment, such as traveling and enjoyment of experience economy (Zhang et al., 2008). Accordingly, our study uses Portrait Value Questionnaire (PVQ) based on the Schwartz theory of ten basic human values (Schwartz, 2006, Schwartz, 2012a,b). We used the PVQ21 items (see Table 1) commonly employed by the European Social Survey (Jowell et al., 2007). These 21 items are supposed to explain the ten basic human values: Tradition, Conformity, Security, Benevolence, Universalism, Self-Direction, Stimulation, Hedonism, Power, and Achievement. According to Schwartz (2012b), these 10 values are hierarchically structured as a circular model as shown in Figure 1. In order to understand how the higher order values are structured and related to the various factors in an explorative manner, all ten factors are included in our analytical process.

#### Travel Experience

As Shamsrizi et al. (2020) reported, individuals’ previous travel experience may affect not only their behavioral intentions for traveling, but also their risk perception, knowledge and health protective behavior against COVID-19. Therefore, previous travel experiences (foreign business, foreign leisure, and domestic leisure) in the past 2 years were questioned.

#### COVID-19 Experience

Zhong et al. (2020) state that “individuals who had direct experience with COVID-19 may have different perspectives on the disease from the public” (Zhong et al., 2020, p2). Considering this, one question asked individuals’ status of the COVID-19 experience (see Table 1).

#### COVID-19 Knowledge

Several previous works (Faasse and Newby, 2020; Motta et al., 2020; Muto et al., 2020; Pagnini et al., 2020; Zhong et al., 2020) addressed individuals’ knowledge related to COVID-19 that affected their health risk perception. In the context of the cross-cultural study addressing East Asians and Europeans, what information was communicated from the media and authorities to the public and what knowledge was acquired by the public may be one of the key factors associated with their risk perceptions and health-protective behaviors. Accordingly, respondents were asked to select “true” or “false” to the six statements defined in Table 1. Some of the questions such as “the virus survive for days outside the body in the open air” and “wearing masks will prevent being infected” may be considered as culturally dependent in nature (Nakayachi et al., 2020).

#### COVID-19 Risk Perception

As a large number of the reviewed works (e.g., Ding et al., 2020; Mainous, 2020; McFadden et al., 2020; Pagnini et al., 2020; Peres et al., 2020; Seale et al., 2020) addressed, the level of risk perception is an important factor that has an impact on individuals’ anxiety and concerns about the COVID-19 pandemic in general. Accordingly, the respondents were asked to indicate to which extent they agree or disagree with the three statements (see Table 1).

#### Intentions to Enjoy Experience Economy

Individuals’ motivational drivers to enjoy experience economy (Zhang et al., 2008) are considered as conflicting values for “behavioral compliance and prosocial behaviors” (Wolf et al., 2020, p619). In order to measure individuals’ internal tension between these two types of behaviors, the respondents were asked to indicate to which extent they agree or disagree with the two statements about intentions to enjoy experience economy and travel abroad.

#### Attitudes to Local Businesses

In order to understand what makes people concerned about a local community becoming a hotspot of an infectious disease, it is crucial to measure individuals’ attitudes to local businesses. Accordingly, we included four statements: importance of foreign tourists visiting a local community; tourists’ responsible behaviors during their visit; the authorities to restrict international tourism; local businesses’ responsibility to make a local community clean and safe for the tourism. The respondents were asked to indicate to which extent they agree or disagree with the four statements.

#### Attitudes to Health Protective Behaviors

As the previous studies pointed out, individuals’ behavioral change for protecting their health is supposed to control the further spread of infectious diseases (Faasse and Newby, 2020; Pagnini et al., 2020; Shiina et al., 2020; Zhong et al., 2020). The health protective behaviors can be classified based on individuals’ relation to others. Specifically, socially responsible behavior that prevents others from becoming infected; self-protective behavior to protect self by avoiding risks of being infected; and behaviors by other public to minimize the spread of infection. The respondents were asked to indicate to which extent they agree or disagree with the eight statements.

### Data Collection

**FIGURE 2:**
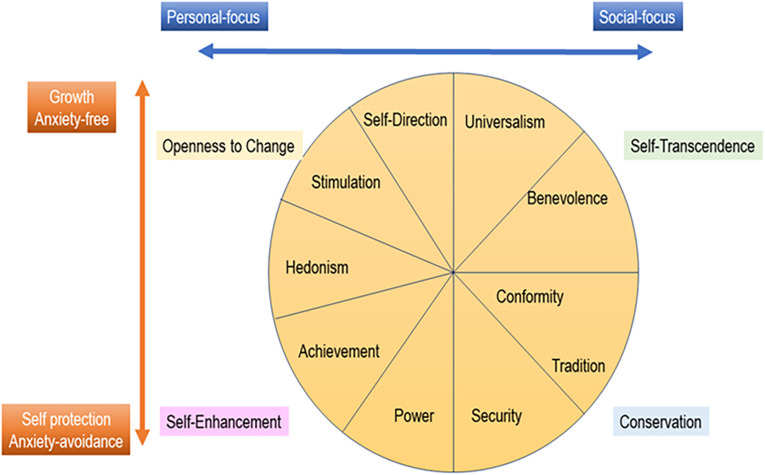
Schwartz theory of ten basic human values (Schwartz, 2012a,b).

Participants from the four countries were recruited from online panels administered by two survey agencies in Denmark and in Japan. Both agencies respectively complied with the GDPR and JIS Q 15001 that protect personal information. A cross-sectional survey was conducted using a self-administered online questionnaire from 10th to 24th of July 2020. Data was collected based on quota sampling representative with regard to gender, age and geography. The target group was defined as male and female in age 18+ years old per country (representative gross sample) who traveled abroad (either business or leisure) at least once or more during the past 3 years. Questionable responses (detected by response time spent for the respective questions) were deleted during the screening process. Accordingly, the total sample (*n* = 4,114) resulted in the four subsets: Denmark (*n* = 1,005), Japan (*n* = 1,091), Italy (*n* = 1,005), and China (*n* = 1,013). Figure 2 overviews the demographic distribution of sample divided into the four subsets. The gender and age distributions of the respective subsets were influenced by the specific sampling criterion that screened participants with previous travel experiences. In particular, the age distribution in the Chinese subset was particularly affected by this criterion. Data is available in the Supplementary Material.

### Data Analysis

**FIGURE 3:**
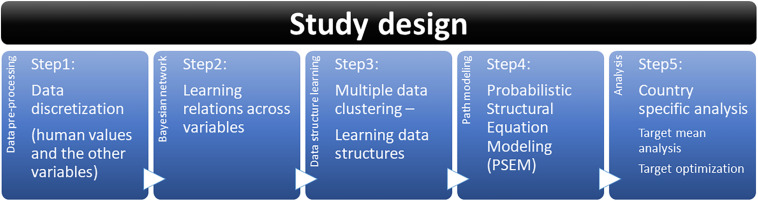
Study design.

Our data analysis is mainly based on the PSEM approach (Conrady and Joufee, 2015, Chapter 8) and consists of five steps as displayed in Figure 3. In the following, we explain the five-step procedure from the data pre-processing to the target optimization that provides unique insights about combinations of factors maximizing or minimizing a mean value of the target variable.

#### Step 1 (Data Pre-processing)

As PSEM based on the Bayesian networks relies on the conditional joint probabilities of the links between variables, we discretized all variables consisting of the continuous- or ordinal categorical data into the discrete data format. Although the ordinal categorical data could be seen as the discrete data format, we employed several discretization criteria considering the probability distributions of the respective question items in order to reduce the computational load of the Bayesian networks and to make the interpretation of the results meaningful.

##### Discretization of the ten basic human factors

Whereas our work is characterized as pragmatic data-driven knowledge discovery, Schwartz’s theory of basic human values (Schwartz, 2006, Schwartz, 2012a,b) is a well-established construct used in various cross-cultural studies. In order to make the smooth interpretation of the value theory and reduce the computational load of the Bayesian network analysis, the PVQ 21 items were reduced to ten factors representing the respective ten basic values. For computing the ten factor scores cross-culturally, we conducted a Multi-Group Confirmatory Factor Analysis (MGCFA) using the (Cross-Cultural) Multi-Group Invariance Testing package in R developed by Fischer and Karl (2019). The fit performance of the configural model reported: Comparative Fit Index (CFI) = 0.925, Tucker-Lewis Index (TLI) = 0.890, Standardized Root Mean Square Residual (SRMR) = 0.047, and Root Mean Square Error of Approximation (RMSEA) = 0.063. The scores were within the acceptable ranges according to Bentler and Hu (1998). Table 2 further indicates that the metric invariance was within the acceptable range, whereas the scalar invariance had to be rejected (CFI. Delta: 0.081 and RMSEA. Delta: 0.24) (Fischer and Karl, 2019). Although this indicated that the direct comparison of means across countries was not defensible, we considered scalar variance to be negligible in the discretization process. Accordingly, the ten factor scores computed for the respective respondents from the four countries served as raw data that were discretized into four levels by setting common thresholds [−0.75, 0, 0.75] for the subsequent Bayesian network analysis.

**TABLE 2:** Fit performance of the multi-group invariance testing.

	**Degree of Freedom (DF)**	**AIC**	**BIC**	**Chi-Square**	**Chi-Sq. difference**	**DF diff.**	***P*-value (>Chi-Sq.)**	**CFI**	**RMSEA**	**CFI. Delta**	**RMSEA. Delta**
Configural	576	256573	259311	2969.2				0.925	0.063	NA	NA
Metric: loadings	609	256662	259190	3123.9	154.69	33	<0.00**	0.921	0.063	0.004	0
Scalar: intercepts	642	259196	261515	5723.8	2599.87	33	<0.00**	0.84	0.087	0.081	0.024
Means	672	261433	263562	8020.4	2296.66	30	<0.00**	0.769	0.102	0.071	0.015

“**” means that the significance level is “0,001”.

##### Discretization of variables

For conducting meaningful interpretation of the explorative analysis, probability densities of the variables have to be taken care. For example, responses to the variable, “*intention to travel abroad*” were concentrated in the range between 1 and 4, while responses to the variable, “*avoid larger groups to avoid risks of infection*” were concentrated in the range between 4 and 7. Accordingly, the seven-point Likert levels were manually merged into the five levels to make balanced response distributions guided by the probability density function of BayesiaLab (Conrady and Joufee, 2015, p36). Similarly, three variables about respondents’ travel experiences were reduced from five levels to three levels using the same technique. Table 3 overviews the discretization criteria of the all variables. During the process of data discretization and import, missing data was treated with the structural EM algorithm.

**TABLE 3:** Data discretization (thresholds and ranges).

	**Threshold**	**Range**	
Travel experience: (business, leisure, domestic)	≤1	1	1
	≤2.5	2	2
	>2.5	3	5
Covid19 risk perception (three items)	**Threshold**	**Range**	
	≤2.5	1	2
	≤3.5	3	3
	≤4.5	4	4
	≤5.5	5	5
	>5.5	6	7
Attitudes to enjoy experience economy: EE	**Threshold**	**Intervals**	
	≤3.5	1	3
	≤4.5	4	4
	≤5.5	5	5
	≤6.5	6	6
	>6.5	7	7
Attitudes to enjoy experience economy: Travel	**Threshold**	**Intervals**	
	≤2.5	1	2
	≤3.5	3	3
	≤4.5	4	4
	>4.5	5	7
Attitudes to avoid risk of COVID-19 infections Attitudes to local businesses Attitudes to responsible behaviors Attitudes to self-protective behaviors Attitudes to public behavior responsible Concern about the hot-spot	**Threshold**	**Intervals**	
	≤3.5	1	3
	≤4.5	4	4
	≤5.5	5	5
	≤6.5	6	6
	>6.52	7	7
Personal value priorities (ten factor scores)	**Threshold**	**Intervals**	
	≤−0.75	−3.0	−0.75
	≤0	−0.75	0.0
	≤0.75	0.0	0.75
	>0.75	0.75	3.0

#### Step 2 (Learning Relations Across Variables)

In our analysis, we used a comprehensive dataset consisting of 47 variables and^1^ respondents from all four countries (*n* = 4,114). Among the 47 variables, the target variable “*individuals’ anxiety about a hotspot*” and a break-out variable “countries” were excluded during the process of learning a Bayesian network structure. The PSEM procedure first learned a network consisting of 45 nodes representing variables of interests and directed links representing causal dependencies among variables. In other words, in a directed link from a parent node A to a child node B, the dependency of the child node B was quantified as a conditional probability table given by a parent node B. To learn a network structure, BayesiaLab used heuristic search algorithms to find a local optimum, while it used various learning algorithms to search spaces and/or strategies. The best performing network structure was selected based on the Minimum Description Length (MDL) score (Conrady and Joufee, 2015, p209–214) that computed the best trade-off between the number of bits representing the Bayesian network and the number of bits representing the dataset given the Bayesian network. In our study, “Taboo Order learning” performed best to express the structure of the dataset. We validated the quality of the network using data perturbation learning that enabled the addition of random noise to the weight of each observation in the dataset (Conrady and Joufee, 2015, p215).

**FIGURE 4:**
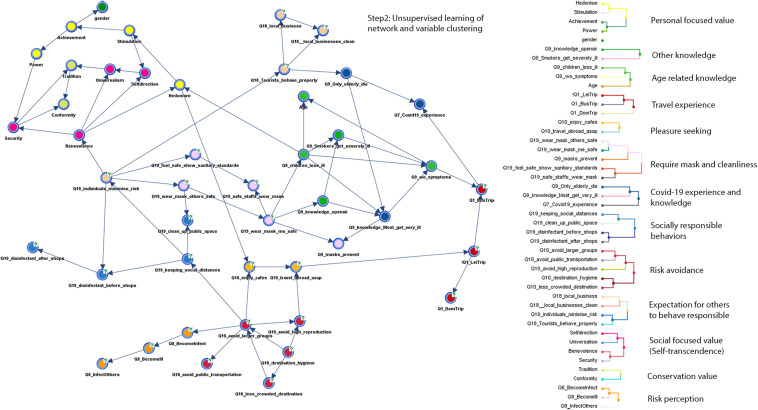
Step 2: An overview of networks (left) and variable clusters (right) acquired from the unsupervised learning.

In order to make the interpretation of the network consisting of 45 variables (nodes) easier and to represent potential theoretical constructs, we used the variable clustering function to group nodes that shared similar data response patterns. The variable clustering algorithm used in BayesiaLab was based on Kullback–Leibler Divergence. In this process, we set the maximum size of the respective variable clusters as five nodes that extracted most meaningful groups of variables corresponding to the number of question items included in the potential constructs explained in section “Measures.” After the initial variable clustering, we further conducted cross-validation to assure the quality of the variable clustering, which resulted in average fit score (purity of the 100 times runs) as 78.7102%. Figure 4 shows the Bayesian network learned in this process. The colors of the nodes indicate the groups of variables identified by the variable clustering procedure. A dendrogram at the right side of Figure 4 overviews a list of variables grouped together. For making the interpretation easier, we assigned a conceptual label for the respective groups of variables.

#### Step 3 (Multiple Data Clustering–Learning Data Structures)

**FIGURE 5:**
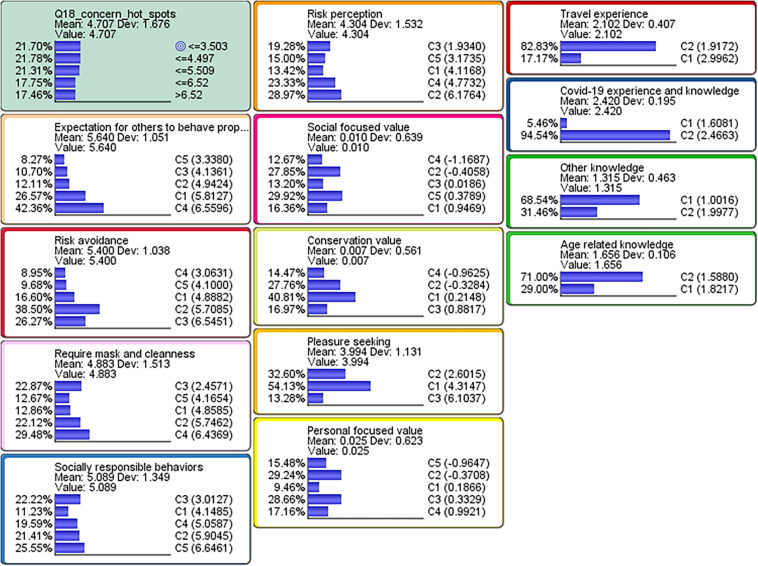
Step 3: Joint probabilities of the respective factors (all four countries).

First, we created a latent variable (i.e., factor) for the respective groups of variables identified in Step 2, and subsequently repeated a process called data clustering process for the respective latent variables. According to Conrady and Joufee (2015, p227), ‘‘this process creates a node that compactly represents the joint probability of distribution defined by the variables of interest.’’ Through this process, 13 discrete latent factors were identified^2^. The results of the multiple data clustering were assessed by the performance indices called “Contingency Table Fit (CTF), which measured the quality of the joint probability distribution representation” defined in Conrady and Joufee (2015, p240). The CTF scores resulted in min. = 63.33% and mean = 84.85% which was consider as reasonable^3^. Figure 5 displays the joint probabilities of the respective factors. In the upper-left box, the probability distribution of responses to the target variable is listed according to the five levels discretized from the seven-point Likert scale in Step 1. The rest of the boxes represent the joint probabilities of the respective latent factors. For each factor, the multiple data clustering algorithm in BayesiaLab created two to five discrete levels of data clusters where observations (respondents) with similar response patterns were grouped together.

#### Step 4 (Probabilistic Structural Equation Modeling: PSEM)

**FIGURE 6:**
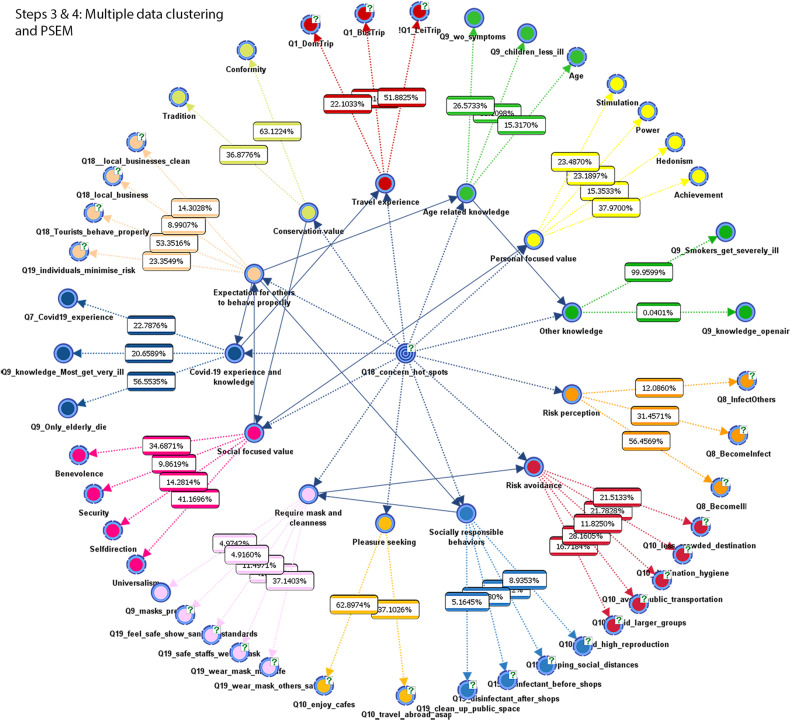
Step 4: Overall links between target node and the 13 latent factor variables as well as links across the 13 latent factor variables obtained by PSEM.

Finally, the target variable excluded in Step 2 was reintroduced in the Bayesian network consisting of the 13 latent factor variables and their manifest variables. At the final stage, PSEM learned an additional network that connected the target node and the 13 latent factor variables without losing the existing network structure between the respective latent factor variables and their manifest variables. In order to maintain these respective relations, the target node as a parent node was first linked manually with the respective 13 latent factor nodes as a child. After confirming these relations, we run the unsupervised learning algorithm called Taboo learning “that can learn a new structure on top of an existing network structure” (Conrady and Joufee, 2015, p249). In this way, the final network displayed in Figure 6 was generated. Figure 6 illustrates the overall links between the target node and the 13 latent factor variables, links between the 13 latent variables and their manifest variables as well as dependency links between the 13 latent variables.

#### Step 5 (Country Specific Analysis)

Based on the global Bayesian network created in the previous step, we generated country-specific networks representing the respective four countries by reintroducing a country variable as a break-out node. These country-specific networks enabled the analysis of the total effects of the 13 latent factor variables on the target variable based on the “parameter estimation” algorithm. For computing the total effects, BayesiaLab recomputed, for the respective country-specific networks, “the values associated with each state of the discretized nodes based on the respective subset of data” (Conrady and Joufee, 2015, p267). Using these four country-specific networks, the Bayesian network approach enabled us to conduct two types of analysis: target mean analysis and target optimization. Selected results of step 5 are presented below in section “Results.”

## Results

**FIGURE 7:**
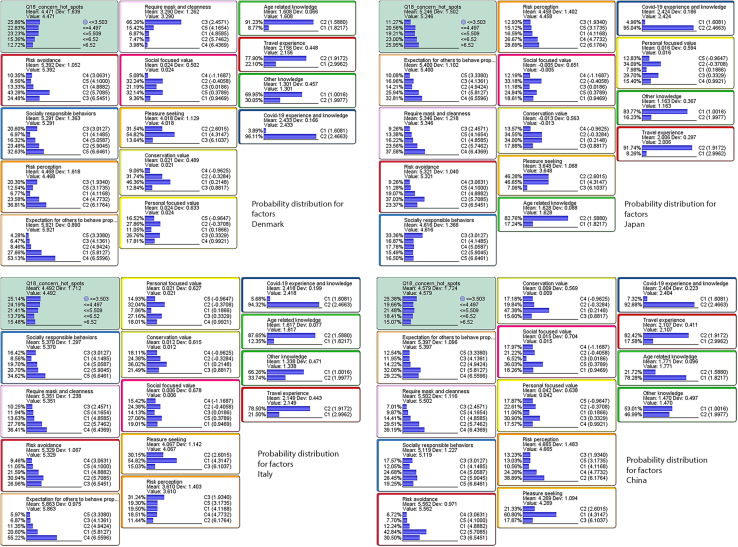
Step 5: Country-specific probability distributions of the target variable and the 13 latent factor variables.

Figure 7 overviews the probability distributions of the target variable and the 13 latent factor variables for the four countries. For each country, the order of the conditional probability tables for the respective factors displayed in the four plots is consistent with the sizes of total effects on the target variable. The plots demonstrate that the probabilistic proportion of respondents who were less concerned about their local community becoming a hotspot of an infectious disease was generally dominant in Denmark, Italy, and China. On the other hand, the probabilistic proportion of respondents who were more concerned about a hotpot was dominant in Japan. The country-specific plots further indicate that ‘‘expectation for others to behave properly’’ and ‘‘require mask and cleanliness’’ were the most important factors affecting ‘‘*individuals’ anxiety about a hotspot*’’ in both Japan and China, while ‘‘risk avoidance’’ and ‘‘socially responsible behaviors’’ were the most important factors in Denmark and Italy, respectively^4^.

### Target Mean Analysis

**FIGURE 8:**
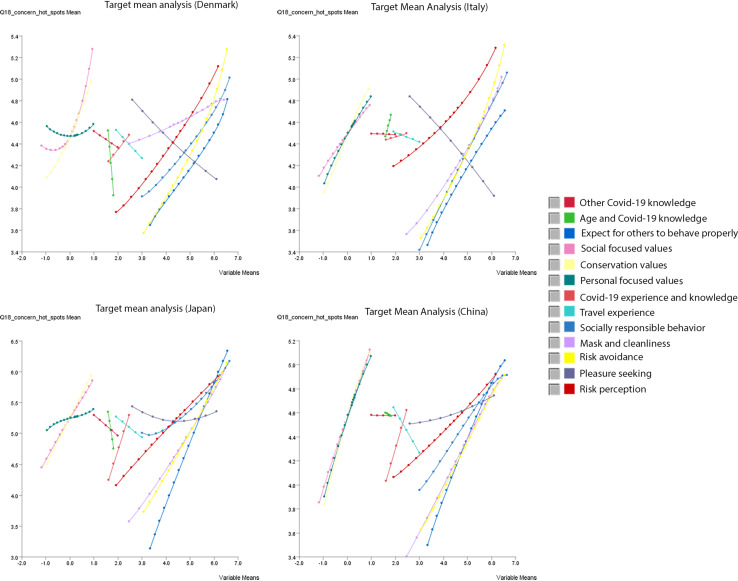
Country-specific target mean analysis: response curves of the target variable (Y-axis) as a function of the values of the 13 factor variables (X-axis).

Figure 8 further^5^ depicts the response curves of the target variable (*Y*-axis) as a function of the values of the 13 factor variables (*X*-axis), i.e., *Y* = *f*(*X*). In the four plots in Figure 8, the ranges of mean values on the *X*-axis differed across the 13 factor variables depending on what manifest variables were connected to the respective factors. Specifically, the factor variables consisting of the “*knowledge*” (variables representing true-false questions combined with age or COVID-19 experience) were placed in the range between 1 and 3, the “*personal values*” consisting of the Schwartz 10 values represented by the factor scores in the range between −2 and 1, and the rest of factor variables in the range over 1 on the *X*-axis. The four plots in Figure 8 illustrate that the shapes of functions representing the 13 factors differed across the four countries. For example, all three personal value factors (named as “personal-focused values,” “social-focused values,” and “conservation values”) affected the target variable in China and Italy, meaning that respondents with negative scores on all three factors were less concerned about the hotspot, while those who were positive to all three value-factors were more concerned about the hotspot. On the other hand, for Denmark and Japan, while “social-focused values” and “conservation values” in general indicated effects on the target variable, “personal-focused values” did not show a strong effect on the target variable.

Similarly, the factors representing the COVID-19 knowledge indicated notable patterns in Figure 8. For example, the factor named as “other COVID-19 knowledge” consisted of two variables: “smokers get very ill” and “the virus survives for days outside the body in the open air.” Figure 8 demonstrates that people who answered “true (=1)” for these questions had a higher probability of indicating higher concern about the hotspot in Denmark and Japan. However, this factor did not show an effect on the target variable in Italy and China.

Another noteworthy phenomenon observed in Figure 8 is that the “pleasure-seeking” factor consisting of manifest variables referring *intentions to enjoy experience economy* and *to travel abroad* had a negative impact on the target variable in Denmark and Italy, whereas its effects were relatively flat in Japan and China. This indicates that, in Denmark and in Italy, people who expressed higher intentions for the pleasure-seeking had higher probabilities of being less concerned about the hotspot, and vice versa. On the other hand, in Japan, disregarding the level of intentions for the pleasure-seeking, the level of concerns about the hotspot stayed around 5.5 (at the level between *somewhat agree* and *agree*). Chinese were similar to Japanese. However, their level of concerns stayed around 4.6–4.8 (at the level between *neutral* and *somewhat agree*).

### Target Optimization

The target optimization of BayesiaLab “performs inference over all possible states of all nodes in a network” (Conrady and Joufee, 2015, p.44). and searches for combinations of factors that maximize or minimize a target mean value. In other words, BayesiaLab searches for sets of evidence based on a so-called “probabilistic evidence” to optimize the mean value (Conrady and Joufee, 2015, p.277). Due to space limitations, we focus on a limited number of selected optimization scenarios identified for Denmark and Japan in the following.

#### Target Maximization

**FIGURE 9:**
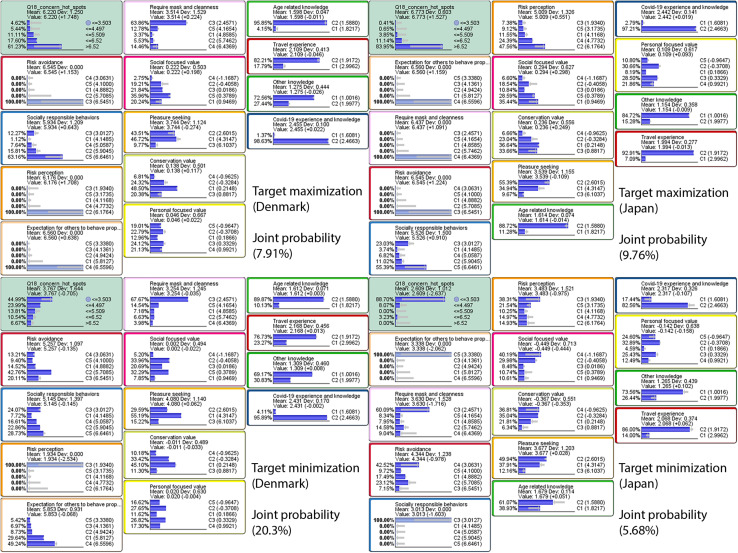
Target optimization scenarios: maximization (upper part) and minimization (lower part) for Denmark (left) and Japan (right).

The two upper plots in Figure 9 show the target maximization scenarios inferred for Denmark and for Japan. Compared to the original probability distributions shown in Figure 7, the probability distributions of the target variable had heavier weights on the higher response category, >6.52 (corresponding to “*strongly agree*”) for the both plots respectively representing the maximization scenarios for Denmark and Japan.

The Danish maximization scenario in the upper-left plot shows that in order to increase the mean value of the target variable +1.748 point to 6.220 (“*agree*” or above), respondents were supposed to belong to the highest score groups for the next three factors: “risk perception” (6.545); “risk avoidance” (6.176); and “expect others to behave properly” (6.559). In this Danish maximization scenario, the probabilities of respondents to belong to higher score groups also increased for other factors such as “socially responsible behaviors,” “require mask and cleanliness,” and “social-focused values,” while the probability to belong to lower score group increased for the “pleasure-seeking” factor. The overall joint probability of this scenario was 7.91%.

The upper-right plot in Figure 9 exhibits the target maximization scenario for Japan. In the best scenario for Japan, the mean value could increase to 6.773 (close to the highest “*strongly agree*”) if a respondent was in the highest score groups for the next three factors: “expectation for others to behave properly” (6.560); “require mask and cleanliness” (6.437); and “risk avoidance” (6.545). In this best scenario, the probabilities of respondents to belong to higher score groups also increased for other factors such as “social responsible behaviors,” “risk perception,” “social-focused values,” and “conservation values,” while the probability to belong to lower score group increased for the “pleasure-seeking” factor. The overall joint probability of this scenario was 9.76%.

#### Target Minimization

In contrast to the previous maximization results, the two lower plots in Figure 9 show the target minimization scenarios for Denmark and for Japan.

For Demark, when a respondent was in the lowest score group of the factor “risk perception” (1.934), the target mean decreased to 3.767 corresponding to the level between *somewhat disagree* and *neutral*. The overall joint probability of this scenario was 20.3%.

On the other hand, the minimization scenario for Japan decreased the mean value of the target value to the level of 2.609 corresponding to the level between *disagree* and *somewhat disagree*. To satisfy this scenario, a respondent was supposed to the lowest score groups for the next two factors: “expectation for others to behave properly” (3.380) and “social responsible behaviors” (3.013). In addition, the probabilities of respondents to belong to the lowest score groups substantially increased for other factors such as “require mask and cleanliness,” “risk avoidance,” “risk perception,” “social focused value,” and “conservation values.” Although the probabilities to belong to the lowest score group also increased slightly for “personal-focused values” and “pleasure-seeking,” the overall distributions for higher score groups were somewhat maintained. In addition, the Japanese minimization scenario indicated that the probability distributions for the knowledge-related factors changed noticeably compared to the other scenarios. The overall joint probability of this scenario was 5.68%.

## Discussion

This article addressed cultural differences in factors affecting a target variable: *individuals’ anxiety about a local community becoming a hotspot of infectious diseases*. As we have chosen a pragmatic approach to understand the reality of the COVID-19 crisis, this section focuses on interpreting the results presented in section “Results.” Although the overall data analysis yielded numerous findings across the four countries, we limit our discussion to the main findings due to space limitations.

### Japanese Were More Concerned About the Local Community Becoming a Hotspot of Infectious Diseases

The results demonstrated that the Japanese population was generally more concerned that the local community will become crowded by foreign tourists and potentially become a hotspot of an infectious disease compared to the rest of the countries. This may be a general reaction of local residents to the recent substantial increase in the inbound tourism that attracted over 30 million foreign visitors in 2019 having been accelerated by the promotion of the Tokyo 2020 Olympic Games originally planned in July--August 2020^6^. The incident of the COVID-19 epidemic in China might have triggered the Japanese population’s anxiety further supported by the fact that, among these increased inbound tourists, over 25% of visitors originated from China. The results observed in our study may be an indication that the recent increase of the inbound tourists from China in combination with the incident of the Diamond Princess Cruise ship made the Japanese population aware about the potential risks of over-tourism that may trigger potential risks of spreading infectious diseases^7^. The characteristics of Japanese being more concerned about the local community becoming a hotspot of infectious diseases might be a reflection of the fact that the factors with highest impact on the target variable for the two East Asian countries were “expectation for others to behave properly” and “require mask and cleanliness.” These two factors imply their expectation for others to comply with the social norm. However, they might also be aware that it is challenging to control behaviors of foreign tourists visiting their local community.

### Relation Between the Personal Value Priority/Knowledge About COVID-19 and Individuals’ Anxiety About a Hotspot Depends on the Severity Level of the Infections at the Time of the Survey Implementation

Our results presented some situational differences in the relation between personal value priorities and *individuals’ anxiety about a hotspot*. Applying the quasi-circumplex model (Figure 2) of the Schwartz theory of ten basic values, Wolf et al. (2020) explains that individuals prioritizing the social-focused (e.g., socially responsible) and conservation (e.g., family security) values will likely comply with the COVID-19 guidelines, while individuals prioritizing the personal-focused values (e.g., freedom and ambition) will be less compliance with the COVID-19 guidelines. From this point, individuals prioritizing the social-focused and conservation values may likely be concerned about a local community becoming a hotspot of an infectious disease for the sake of society and their in-group community, while those prioritizing the personal-focused values may not. In our results, the patterns observed in Denmark and Japan were rather rational in this respect. Disregarding the level of priorities in the personal-focused values, the anxiety level of Danish and Japanese populations was maintained around medium-low level in Figure 8. However, this rationale was not applicable to Italy and China. Even those who prioritized personal-focused values (e.g., freedom and ambition) expressed concerns about their local community become a hotspot of an infectious disease in Italy and China. This could be explained by the fact that, at the time of survey implementation in the middle of July, Italy and China were already severely hit by the COVID-19 infection.

The knowledge about the COVID-19 possessed by individuals may be another factor associated with *individuals’ anxiety about a hotspot*. The existing literature (Faasse and Newby, 2020; Muto et al., 2020; Pagnini et al., 2020; Shiina et al., 2020; Zhong et al., 2020) reported that the level of knowledge about COVID-19 correlated with individuals’ risk perception. Accordingly, it may be expected that a level of knowledge possessed by individuals will affect their level of anxiety about a hotspot. Our results exhibited this tendency for Japan and Denmark. However, the anxiety level of Italian and Chinese populations was rather stable, independently of whether their knowledge about COVID-19 was right or wrong in most of the questions.

These two observations indicate that the severity level of the infections at the time of the survey implementation for the four countries may have moderated the relations between the personal value priority/knowledge about COVID-19 and the level of anxiety about a hotspot among the Italian and Chinese populations. For this reason, the effects of the personal-focused values and the knowledge may not have exhibited observable effects in the case of Italy and China.

### Relation Between the Pleasure-Seeking and Individuals’ Anxiety About a Hotspot Differed Between East Asia and Europe

Another noteworthy finding is that there was a cultural difference between East Asian and European populations regarding the relation between the “pleasure-seeking” factor and *individuals’ anxiety about a hot spot*. Earlier studies implied that individuals’ motivational drivers to enjoy the experience economy (Zhang et al., 2008) is considered as conflicting values for “behavioral compliance to government guidelines and promoting prosocial behaviors” (Wolf et al., 2020, p618). Individuals prioritizing in enjoying the experience economy may therefore be less concerned about a local community becoming a hotspot of infectious diseases in general. However, such a phenomenon was observed only in Denmark and Italy, while the level of anxiety about a hotspot remained high despite the level of intention to enjoy the experience economy among the Japanese and Chinese populations.

This separation between East Asian and European may reflect “interdependent self-schema” and “independent self-schema,” respectively, in collectivistic and individualistic societies (Markus and Kitayama, 1991). As the study on the Japanese population by Parady et al. (2020) suggested, “the perception of self-restriction of others” may play an important role when a person is based on the interdependent self-scheme. The statement by Uchida et al. (2004) explains that Westerns (Europeans)’s happiness relies on “positivity of the personal self” while Eastern Asians’ happiness is “a state that is contingent on social harmony and, thus, on a balance among different selves in a relationship (p227).” From this view, the enjoyment of the experience economy may be construed as “a state contingent on the positivity of the personal self” for Europeans. On the other hand, for East Asians, the protection of a local community (i.e., in-group) from infectious disease may have higher priority than the positivity of the personal self, because their happiness relies on a state that ensures social harmony and a balance among different selves in a relationship.

### Combinations (Chains) of Factors Affecting the Target Variable

As revealed in Figures 7, 9, for East Asians, the most important factors affecting their “*anxiety about a hotspot*” were “expectation for others to behave properly” and “require mask and cleanliness.” In short, East Asians seemed to value others to comply with a social norm. On the other hand, Europeans had more emphasis on the factors such as “risk avoidance,” “risk perception,” and “socially responsible behaviors” that address the self-protection and expressions. Our study extracted the best scenarios that maximize or minimize the mean value of the target variable: *individuals’ anxiety about a hotspot* to investigate further details.

The combinations of factors explaining the optimization scenarios in Figure 9 signposted that the psychological tension between the pleasure-seeking versus the concerns about their local community becoming a hotspot was clearly observed among the Danish population. In the scenario of maximizing the mean value of the target variable, “*individuals’ anxiety about a hotspot*,” the mean value of the pleasure-seeking factor decreased 0.274 point, while in the scenario of the minimization it increased 0.062 point. Similarly, for the maximization, the increase of the mean value for the “personal-focused values” was moderate (0.022), whereas for the minimization, the increase was substantial (0.630). As the mean value of the anxiety increased, the mean value of the self-restricted behaviors also increased, and vice versa. The result clearly confirmed that the psychological tension between opposing human factors, i.e., free enjoyment versus the self-restriction existed among the Danish population. However, the same pattern was not observed among the Japanese population. This result once again confirms the work by Uchida et al. (2004) indicating that the enjoyment of the experience economy may be construed as “a state contingent on the positivity of the personal self” for Danes. On the other hand, for Japanese, the level of anxiety about their community becoming a hotspot seemed to correlate with their level of compliance with a social norm. In the maximization scenario, the mean values of the social-focused values and the conservation values, respectively increased 0.298 and 0.249 points, while these scores decreased 0.444 and 0.353 points, respectively in the minimization scenario. This could be explained by the fact that the anxiety about a local community becoming a hotspot may be closely connected with a protection of the in-group community. For protecting the in-group community, the compliance with the social norm may play an important role among the Japanese population. However, as Muto et al. (2020) reported, a specific segment of people is not willing to comply with the social norm. The minimization scenario demonstrated this phenomenon in Japan.

Another important note is that the distance between the maximized mean value (mean: 6.773) and the minimized mean value (2.609) was substantial in the Japanese population. Considering that the survey response style by Japanese is expected to be generally moderate due to the reference effect (Heine et al., 2002), the extreme positive or negative responses to the survey was unexpected. It seems that their reactions to this specific COVID-19 topic was exceptional. Once again, the reactions to the COVID-19 seemed to be associated with the social inequality issue in Japan as indicated by Muto et al. (2020).

### Managerial Implication

The findings highlighted the importance of understanding the contextual differences between psychological factors and the target variable. The severity of the infections at the time of survey implementation in the four countries was one of the contexts that may have moderated the relations between the personal value priority/knowledge about COVID-19 and the level of anxiety about a hotspot in particular among the Italian and Chinese populations. The cultural context distinguishing the interdependent and independent self-schemas was another important context that may have moderated the relations between pleasure-seeking behaviors and the anxiety about a hotspot. Finally, our target optimization study also identified that the population within a country was heterogeneous. To explain the high-level of anxiety or the low-level of anxiety about a hotspot, various combination of factors were involved. Especially, the combinations of factors implied that there was a tension between the pleasure-seeking and the behaviors involving self-restrictions. However, these tensions can be culture-dependent. From a managerial viewpoint, a tug of war between fast economic recovery and firm control of further virus-spread is one of the critical COVID-19 challenges. To achieve the prevention of COVID-19 while maintaining the experience economy, policy makers of the public health and the experience economy industry must understand diverse scenarios explaining about the interpersonal tension between the pleasure-seeking versus the health perception, risks and protective behaviors. The insights identified in our study could help policy makers to consider expected cultural differences and individual differences when they develop a measure to solve the COVID-19 dilemma between the public health and the recovery of the experience economy.

### Limitations and Future Directions

Our survey was conducted in the middle of July 2020. At that time, the four countries implemented different measures in terms of border control and tourism promotions. For Japan and China, their national borders were closed to foreign tourists. Hence, their experience economy depended mostly on the domestic market. On the other hand, in Europe, national borders were gradually opened during the summer vacation period (June–August) in order to promote their local economy relying on international tourism. This difference between the East Asian- and the European measures may be considered as a possible bias that influenced subjects’ responses to our survey. On the other hand, this political-economic situation in East Asia and Europe has some interesting implications. The European countries who allowed their populations to travel around Europe had to deal with the diversity of foreign tourists visiting their local communities. By contrast, both in China and in Japan, the experience economy could rely on domestic demand to a certain extent. This means that policy makers in these countries were able to restrict entries of foreign visitors including their own nationals residing abroad and focus on controlling their own populations who were inclined to care for the social norm and their own in-group community. An interesting question is how these countries will deal with foreign visitors who seek “the positivity of the personal self” (Uchida et al., 2004, p227) once their borders will be opened for foreign tourists in the future. They may suddenly need to understand the cultural sensitivity of foreign visitors.

Our study has chosen a pragmatic approach employing PSEM based on the principle of Bayesian networks. The applied PSEM approach demonstrated some insights in which the conventional structural equation modeling (conventional SEM) has limitations. First of all, the conventional SEM requires researchers to define a theoretical model (hypotheses) prior to the cross-cultural analysis. When it comes to the cross-cultural analysis, the conventional approach employs the multi-group SEM (MGSEM) which also requires the establishment of the configural- and scalar invariance to compare the assumed underlying psychological construct (Fischer and Karl, 2019). We expect that this strictness in the conventional approach would have restricted our comprehensive understanding of the phenomena observed in the four countries. In other words, the application of Bayesian network analysis enabled us to include relatively large number of variables and possible theoretical constructs without strictly defining a specific model, to visually inspect probability distributions of responses to the respective variables and factors, to simulate what combinations of factors impacted on increasing or decreasing the mean value of the target variable and to estimate what proportion of population fell under specific optimization scenarios.

One of the important notes is that our Bayesian network analysis relied on discrete data, meaning that all ordinal categorical data was further discretized into smaller numbers of discrete categories. This means that the results of the analysis largely depended on how we discretized our data. Although the skewness and kurtosis were manually handled based on the density distributions, the process of discretization could be improved and systematically defined (Glückstad et al., 2020; Thiem and Duşa, 2013) in future works. Another note is that, in our analysis, we integrated age and gender as demographic variables, and COVID-19 experiences as a background variable. During the Bayesian network leaning process, these variables (e.g., gender, age) were eliminated or merged with other constructs. In future research, it may be an idea to treat these variables as “break-out” variables, similar to the way we treated the country variable. Finally, the PSEM approach enabled the extractions of potentially uncovered cultural differences and conceptual relations that could help researchers to develop interesting hypotheses. An obvious future research study would be to develop a theoretical model and hypotheses based on our findings and test them by the conventional SEM approach.

A final remark is that the data-driven approach used in this paper, i.e., using Bayesian network techniques to infer a casual network from data by associating variables with a conditional probability, has a potential limitation. The fact that the structure of the Bayesian network was learnt from data rather than from knowledge of causality means that the approach can be categorized as learning from association, which is the first rung on the Ladder of Causation according to Pearl and Mackenzie (2018). Hence, it is not reasonable to assume that the learned Bayesian network structure can be used for intervention and counterfactual reasoning (the second and third rungs, respectively, in the Ladder of Causation), whereas a Bayesian network structure that incorporates causal knowledge could.

## Data Availability Statement

The original contributions presented in the study are included in the article/Supplementary Material, further inquiries can be directed to the corresponding author/s.

## Ethics Statement

Ethical review and approval was not required for the study on human participants in accordance with the local legislation and institutional requirements. The patients/participants provided their written informed consent to participate in this study.

## Author Contributions

FG designed the overall study, the questionnaire and the data analysis procedure and also conducted the literature review, the entire data analysis, the interpretation of the results, and the writing of the overall manuscript. PA contributed to the design of the questionnaire, the literature review as well as the review and revision of the introduction, the research strategy, and the discussion sections. UW contributed to the critical review and revision in particular sections addressing the method design and results of the data analysis. MM contributed to the review of the manuscript. All authors contributed to the article and approved submitted version.

## Conflict of Interest

The authors declare that the research was conducted in the absence of any commercial or financial relationships that could be construed as a potential conflict of interest.

## Footnotes

1 Full details of the fit score is presented in Appendix 2 of the Supplementary Material.

2 Although we were aware that some variables were grouped into a factor that was not assumed in section “Personal Values” describing the Schwartz Theory, we did not modify the variable clusters, as we chose to conduct the data-driven explorative approach through the entire procedure.

3 Full details of the fit score is presented in Appendix 3 of the Supplementary Material.

4 Country-specific probability distributions of the respective manifest variables affecting the target node can be found in Appendix 4 of the Supplementary Material.

5 Country-specific target mean analyses for the manifest variables can be found in Appendix 5 of the Supplementary Material.

6 https://statistics.jnto.go.jp/

7 https://en.wikipedia.org/wiki/COVID-19_pandemic_on_Diamond_Princess
