# Supplementary material for: Cross-Cultural Bayesian Network Analysis of Factors Affecting Residents’ Concerns About the Spread of an Infectious Disease Caused by Tourism
Source: Front Psychol. 2021 Jun 7;12:635110. doi: 10.3389/fpsyg.2021.635110 (PMC8215548; doi:10.3389/fpsyg.2021.635110)
Supplement: Supplementary file 1 [file Data_Sheet_1.ZIP › SpplementalMaterials/Supplementary materials.pdf]

Supplemental materials

Appendix1 - Step1: Multi-group Confirmatory Factor Analysis (MGCFA)

Method based on Fischer & Karl (2019) A Primer to (Cross-Cultural) Multi-Group Invariance Testing Possibilities in R, Frontiers in Psychology, Vol.10, p.1-18.

Fit performance

Multigroup SEM for PVQ21 (DK, IT, JP, CN)	
> ## Fit inidices of the CFA	
> summary(fit_cfa_country, fit.measures = TRUE,	
+ standardized = TRUE, rsquare = TRUE)	
lavaan 0.6-7 ended normally after 135 iterations	
Estimator	ML
Optimization method	NLMINB
Number of free parameters	432
Number of observations per group:	
JP	1110
CN	1019
IT	1014
DK	1028
Model Test User Model:	
Test statistic	2969.196
Degrees of freedom	576
P-value (Chi-square)	0.000
Test statistic for each group:	
JP	699.718
CN	579.341
IT	881.840
DK	808.297
Model Test Baseline Model:	
Test statistic	32618.813
Degrees of freedom	840
P-value	0.000
User Model versus Baseline Model:	
Comparative Fit Index (CFI)	0.925
Tucker-Lewis Index (TLI)	0.890
Loglikelihood and Information Criteria:	
Loglikelihood user model (H0)	-127854.732
Loglikelihood unrestricted model (H1)	-126370.134
Akaike (AIC)	256573.464
Bayesian (BIC)	259310.577
Sample-size adjusted Bayesian (BIC)	257937.865
Root Mean Square Error of Approximation:	
RMSEA	0.063
90 Percent confidence interval - lower	0.061
90 Percent confidence interval - upper	0.065
P-value RMSEA <= 0.05	0.000
Standardized Root Mean Square Residual:	
SRMR	0.047
Parameter Estimates:	
Standard errors	Standard
Information	Expected
Information saturated (h1) model	Structured

Measurement invariance models:

Model 1 : fit.configural
Model 2 : fit.loadings
Model 3 : fit.intercepts
Model 4 : fit.means

Chi-Squared Difference Test

	Df	AIC	BIC	Chisq	Chisq diff	Df dkff		Pr(>Chisq)				
fit.configu	576	256573	259311	2969.2								
fit.loading	609	256662	259190	3123.9	154.69	33	<	2.20E-16	***			
fit.intercep	642	259196	261515	5723.8	2599.87	33	<	2.20E-16	***			
fit.means	672	261433	263562	8020.4	2296.66	30	<	2.20E-16	***			

Signif.	codes:	0	'***'	0.001	'**'	0.01	'*'	0.05	'.'	0.1	'	'

Fit measures:

	cfi	rmsea	cfi.delta	rmsea.delta
fit.configu	0.925	0.063	NA	NA
fit.loading	0.921	0.063	0.004	0
fit.intercep	0.84	0.087	0.081	0.024
fit.means	0.769	0.102	0.071	0.015

>	equival(cfa	dat	=	mydata,	group	=	ID)				
	CFI	RMSEA	LC	UC	SRMR	GAMMA	MNCI	DELTACFI	DELTARMS	DELTAGAM	DELTAMNCI
Configural	NA	NA	0	0	0.272393	0.604376	0.081192	NA	NA	NA	NA
Metric	0.341253	0.158905	0.156866	0.160954	0.272541	0.675955	0.134396	NA	NA	-0.07158	-0.0532
Scalar	0.279837	0.162778	0.160838	0.164727	0.276394	0.655226	0.098179	0.061416	-0.00387	0.020728	0.036217

Group 1 [JP]:

Latent Variables:

	Estimate	Std.Err	z-value	P(>|z|)	Std.lv	Std.all
Tradition	=~					
TR1	1				0.605	0.51
TR2	1.393	0.094	14.848	0	0.843	0.72
Conformit	=~					
CO1	1				0.887	0.775
CO2	0.94	0.04	23.28	0	0.834	0.715
Security	=~					
SC1	1				0.832	0.716
SC2	0.991	0.05	19.88	0	0.824	0.689
Hedonism	=~					
HD1	1				0.552	0.483
HD2	1.628	0.108	15.129	0	0.899	0.76
Stimulatio	=~					
ST1	1				0.913	0.764
ST2	1.041	0.044	23.821	0	0.95	0.799
Selfdirecti	=~					
SD1	1				0.854	0.7
SD2	1.007	0.046	22.088	0	0.86	0.692
Power	=~					
PO1	1				0.767	0.659
PO2	1.061	0.054	19.706	0	0.814	0.72
Achieveme	=~					
AC1	1				0.926	0.785
AC2	0.889	0.039	22.74	0	0.823	0.716
Universalis	=~					
UN1	1				0.87	0.744
UN2	0.964	0.039	24.574	0	0.839	0.748
UN3	0.972	0.043	22.695	0	0.846	0.694
Benevolen	=~					
BE1	1				0.911	0.764
BE2	1.066	0.039	27.182	0	0.97	0.809

Group2 [CN]:

Latent Variables:

	Estimate	Std.Err	z-value	P(>|z|)	Std.lv	Std.all
Tradition	=~					
TR1	1				0.667	0.529
TR2	1.25	0.077	16.245	0	0.834	0.703
Conformit	=~					
CO1	1				0.791	0.636
CO2	0.664	0.057	11.708	0	0.526	0.435
Security	=~					
SC1	1				0.857	0.673
SC2	1.124	0.051	22.183	0	0.964	0.762
Hedonism	=~					
HD1	1				0.852	0.696
HD2	0.942	0.046	20.538	0	0.802	0.681
Stimulatio	=~					
ST1	1				0.814	0.679
ST2	0.92	0.053	17.432	0	0.749	0.6
Selfdirecti	=~					
SD1	1				0.841	0.679
SD2	0.961	0.046	20.674	0	0.809	0.702
Power	=~					
PO1	1				0.776	0.599
PO2	0.697	0.058	12.095	0	0.541	0.431
Achieveme	=~					
AC1	1				0.869	0.708
AC2	0.911	0.049	18.412	0	0.792	0.621
Universalis	=~					
UN1	1				0.859	0.694
UN2	0.941	0.045	20.736	0	0.808	0.684
UN3	0.912	0.047	19.235	0	0.783	0.633
Benevolen	=~					
BE1	1				0.943	0.767
BE2	0.992	0.039	25.545	0	0.935	0.778

Group3 [IT]:

Latent Variables:

	Estimate	Std.Err	z-value	P(>|z|)	Std.lv	Std.all
Tradition	=~					
TR1	1				0.692	0.475
TR2	1.124	0.088	12.766	0	0.778	0.591
Conformit	=~					
CO1	1				0.856	0.628
CO2	0.901	0.062	14.519	0	0.772	0.557
Security	=~					
SC1	1				0.813	0.602
SC2	0.993	0.067	14.853	0	0.807	0.567
Hedonism	=~					
HD1	1				0.902	0.711
HD2	1.093	0.059	18.55	0	0.986	0.768
Stimulatio	=~					
ST1	1				0.741	0.536
ST2	1.14	0.078	14.57	0	0.845	0.635
Selfdirecti	=~					
SD1	1				0.857	0.663
SD2	0.93	0.056	16.581	0	0.796	0.641
Power	=~					
PO1	1				0.708	0.547
PO2	1.014	0.077	13.185	0	0.718	0.518
Achieveme	=~					
AC1	1				0.89	0.649
AC2	1.022	0.057	18.083	0	0.91	0.717
Universalis	=~					
UN1	1				0.892	0.716
UN2	0.88	0.047	18.827	0	0.785	0.666
UN3	0.957	0.051	18.652	0	0.854	0.66
Benevolen	=~					
BE1	1				0.863	0.704
BE2	0.997	0.051	19.667	0	0.86	0.713

Group4 [DK]:

Latent Variables:

Estimate	Std.Err	z-value	P(>|z|)	Std.lv	Std.all	
Tradition	=~					
TR1	1				0.409	0.318
TR2	1.272	0.125	10.201	0	0.521	0.409
Conformit	=~					
CO1	1				0.581	0.474
CO2	1.179	0.1	11.8	0	0.686	0.539
Security	=~					
SC1	1				0.601	0.475
SC2	1.203	0.12	10.056	0	0.723	0.557
Hedonism	=~					
HD1	1				0.8	0.724
HD2	1.199	0.068	17.654	0	0.96	0.788
Stimulatio	=~					
ST1	1				0.866	0.727
ST2	1.033	0.056	18.356	0	0.895	0.743
Selfdirecti	=~					
SD1	1				0.441	0.326
SD2	1.312	0.13	10.108	0	0.578	0.53
Power	=~					
PO1	1				0.751	0.635
PO2	0.846	0.062	13.674	0	0.635	0.518
Achieveme	=~					
AC1	1				0.969	0.779
AC2	0.88	0.048	18.326	0	0.853	0.655
Universalis	=~					
UN1	1				0.584	0.472
UN2	1.53	0.123	12.409	0	0.893	0.697
UN3	1.382	0.117	11.81	0	0.807	0.609
Benevolen	=~					
BE1	1				0.829	0.715
BE2	1.004	0.055	18.298	0	0.832	0.776

Appendix 2 - Step2: Unsupervised learning of Bayesian network and variable clustering

Unsupervised learning
Taboo Order learning:
Post-Processed with Taboo (157)
Taboo List Size: 10
Missing values treated with Structural EM
Total Weight: 4,114
Initial MDL score: 329,132.768
Final MDL score: 267,590.241
Total learning time: 1m 0s

Data perturbation learning:
Number of Tests: 100, Final Standard Deviation: 0, Algorithm: Taboo Order, Structural Coefficient Standard Deviation: 1, Minimum SC: 0.2, Maximum SC: 1
Missing values treated with Structural EM
Total Weight: 4,114
Initial MDL score: 329,132.768
Final MDL score: 267,590.241
Total learning time: 2h 21m 4s

Variable clustering – cross-validation
Purity of the 100 times runs:

Average Fit Score 78.7102%	
Standard Deviation	5.1262%
Minimum	68.8172%
Maximum	88.9113%

Appendix 3 - Step 3: Multiple Data Clustering

Automatic Selection of the Number of States by Random Walk	
Number of Steps	50
Sample Size	100.0000%
Initial Number of States	2
Maximum Number of States	5
Minimum Cluster Purity	70.0000%
Minimal Cluster Size	1.0000%
Random Number Generator's Seed	31

Result Summary			
**Statistics**	**Min**	**Mean**	**Max**
Number of Factors			13
Number of Clusters	2	3.8462	5
Mean Purity	87.1078%	93.0486%	100.0000%
Contingency Table Fit	63.3264%	84.8536%	100.0000%
Hypercube Cells Per State	2.0000	79.0847	457.7441

Require mask and cleanliness

Performance Indices	
Number of Clusters	5
Mean Purity	89.8206%
Contingency Table Fit	80.1024%
Deviance	2,278.1220
Hypercube Cells Per State	200.2561

Overall Analysis with [Factor_0]											
Node	Mutual Information	Normalized Mutual Information	Relative Mutual Information	Relative Significance	Prior Mean Value	G-test	df	p-value	G-test (Data)	df (Data)	p-value (Data)
Q19_wear_mask_others_safe	1.2483	53.7619%	55.5838%	1.0000	5.0045	7,119.3911	16	0.0000%	7,113.8668	16	0.0000%
Q19_wear_mask_me_safe	1.2025	51.7890%	53.5440%	0.9633	4.8518	6,858.1321	16	0.0000%	6,866.1528	16	0.0000%
Q19_safe_staffs_wear_mask	0.6604	28.4417%	29.4056%	0.5290	5.2045	3,766.3843	16	0.0000%	3,786.1263	16	0.0000%
Q19_feel_safe_show_sanitary_standards	0.4447	19.1541%	19.8032%	0.3563	5.5269	2,536.4781	16	0.0000%	2,547.6161	16	0.0000%
Q9_masks_prevent	0.1290	5.5572%	5.7456%	0.1034	1.3493	735.9161	4	0.0000%	735.9504	4	0.0000%

Other Covid-19 knowledge

Performance Indices	
Number of Clusters	2
Mean Purity	100.0000%
Contingency Table Fit	100.0000%
Deviance	-0.0000
Hypercube Cells Per State	2.0000

Overall Analysis with [Factor_1]											
Node	Mutual Information	Normalized Mutual Information	Relative Mutual Information	Relative Significance	Prior Mean Value	G-test	df	p-value	G-test (Data)	df (Data)	p-value (Data)
Q9_Smokers_get_severely_ill	0.8983	89.8340%	100.0000%	1.0000	1.3145	5,123.4261	1	0.0000%	5,123.4261	1	0.0000%
Q9_knowledge_openair	0.0039	0.3889%	0.4329%	0.0043	1.4064	22.1817	1	0.0002%	22.1817	1	0.0002%

Covid-19 experience and knowledge

Performance Indices	
Number of Clusters	2
Mean Purity	98.0892%
Contingency Table Fit	84.9947%
Deviance	57.7783

Performance Indices	
Hypercube Cells Per State	8.4995

Overall Analysis with [Factor_2]											
Node	Mutual Information	Normalized Mutual Information	Relative Mutual Information	Relative Significance	Prior Mean Value	G-test	df	p-value	G-test (Data)	df (Data)	p-value (Data)
Q9_Only_elderly_die	0.1610	16.1007%	52.7891%	1.0000	1.9193	918.2568	1	0.0000%	918.2568	1	0.0000%
Q9_knowledge_Most_get_very_ill	0.0862	8.6235%	28.2736%	0.5356	1.7965	491.8142	1	0.0000%	491.8142	1	0.0000%
Q7_Covid19_experience	0.0837	8.3734%	27.4537%	0.5201	4.0233	477.5525	4	0.0000%	477.5525	4	0.0000%

Socially responsible behaviors

Performance Indices	
Number of Clusters	5
Mean Purity	89.3493%
Contingency Table Fit	86.8033%
Deviance	1,197.1629
Hypercube Cells Per State	108.5042

Overall Analysis with [Factor_3]											
Node	Mutual Information	Normalized Mutual Information	Relative Mutual Information	Relative Significance	Prior Mean Value	G-test	df	p-value	G-test (Data)	df (Data)	p-value (Data)
Q19_disinfectant_before_shops	1.3311	57.3253%	58.4759%	1.0000	5.0350	7,591.2748	16	0.0000%	7,598.1704	16	0.0000%
Q19_disinfectant_after_shops	1.2880	55.4730%	56.5864%	0.9677	5.0715	7,345.9896	16	0.0000%	7,346.8462	16	0.0000%
Q19_keeping_social_distances	0.5105	21.9872%	22.4285%	0.3836	5.4029	2,911.6402	16	0.0000%	2,918.2914	16	0.0000%
Q19_clean_up_public_space	0.3387	14.5890%	14.8818%	0.2545	5.2264	1,931.9437	16	0.0000%	1,939.0015	16	0.0000%

Risk avoidance

Performance Indices	
Number of Clusters	5
Mean Purity	87.1078%
Contingency Table Fit	73.2391%
Deviance	3,312.8279
Hypercube Cells Per State	457.7441

Overall Analysis with [Factor_4]

Node	Mutual Information	Normalized Mutual Information	Relative Mutual Information	Relative Significance	Prior Mean Value	G-test	df	p-value	G-test (Data)	df (Data)	p-value (Data)
Q10_avoid_larger_groups	0.8219	35.3982%	39.0564%	1.0000	5.5277	4,687.5903	16	0.0000%	4,698.7495	16	0.0000%
Q10_destination_hygiene	0.8050	34.6683%	38.2510%	0.9794	5.4302	4,590.9293	16	0.0000%	4,618.0413	16	0.0000%
Q10_less_crowded_destination	0.7671	33.0367%	36.4509%	0.9333	5.2539	4,374.8758	16	0.0000%	4,369.1818	16	0.0000%
Q10_avoid_high_reproduction	0.5985	25.7740%	28.4376%	0.7281	5.7699	3,413.1119	16	0.0000%	3,405.4349	16	0.0000%
Q10_avoid_public_transportation	0.4942	21.2861%	23.4859%	0.6013	4.8452	2,818.8041	16	0.0000%	2,818.9163	16	0.0000%

Travel experience

Performance Indices	
Number of Clusters	2
Mean Purity	94.5046%
Contingency Table Fit	63.3264%
Deviance	252.3105
Hypercube Cells Per State	8.5491

Overall Analysis with [Factor_5]

Node	Mutual Information	Normalized Mutual Information	Relative Mutual Information	Relative Significance	Prior Mean Value	G-test	df	p-value	G-test (Data)	df (Data)	p-value (Data)

Overall Analysis with [Factor_5]

!Q1_LeiTrip	0.3219	32.1875%	48.6664%	1.0000	2.2181	1,835.7220	2	0.0000%	1,836.2168	2	0.0000%
Q1_DomTrip	0.1753	17.5297%	26.5043%	0.5446	2.4446	999.7551	2	0.0000%	1,001.1551	2	0.0000%
Q1_BusTrip	0.1521	15.2083%	22.9944%	0.4725	1.4629	867.3598	2	0.0000%	863.3501	2	0.0000%

Age related Covid-19 knowledge

Performance Indices	
Number of Clusters	2
Mean Purity	94.3086%
Contingency Table Fit	80.3374%
Deviance	180.8305
Hypercube Cells Per State	8.0337

Overall Analysis with [Factor_6]

Node	Mutual Information	Normalized Mutual Information	Relative Mutual Information	Relative Significance	Prior Mean Value	G-test	df	p-value	G-test (Data)	df (Data)	p-value (Data)
Q9_children_less_ill	0.4606	46.0554%	53.0163%	1.0000	1.2659	2,626.6375	1	0.0000%	2,626.6375	1	0.0000%
Q9_wo_symptoms	0.2621	26.2097%	30.1711%	0.5691	1.1529	1,494.7952	1	0.0000%	1,494.7952	1	0.0000%
Age	0.1883	18.8263%	21.6718%	0.4088	3.3094	1,073.7058	4	0.0000%	1,073.7058	4	0.0000%

Expectation for others to behavior responsible

Performance Indices	
Number of Clusters	5
Mean Purity	88.6333%
Contingency Table Fit	86.3074%
Deviance	1,024.4874
Hypercube Cells Per State	107.8843

Overall Analysis with [Factor_7]											
Node	Mutual Information	Normalized Mutual Information	Relative Mutual Information	Relative Significance	Prior Mean Value	G-test	df	p-value	G-test (Data)	df (Data)	p-value (Data)
Q18_Tourists_behave_properly	1.1750	50.6064%	57.4898%	1.0000	5.8318	6,701.5351	16	0.0000%	6,709.1567	16	0.0000%
Q19_individuals_minimise_risk	0.7067	30.4351%	34.5748%	0.6014	5.9399	4,030.3573	16	0.0000%	4,026.1381	16	0.0000%
Q18__local_businesses_clean	0.6553	28.2232%	32.0621%	0.5577	5.2970	3,737.4458	16	0.0000%	3,741.9844	16	0.0000%
Q18_local_business	0.4486	19.3188%	21.9465%	0.3817	4.9254	2,558.2838	16	0.0000%	2,562.1357	16	0.0000%

Personal focused value

Performance Indices	
Number of Clusters	5
Mean Purity	94.8699%
Contingency Table Fit	91.2824%
Deviance	1,168.8308
Hypercube Cells Per State	46.7366

Overall Analysis with [Factor_8]											
Node	Mutual Information	Normalized Mutual Information	Relative Mutual Information	Relative Significance	Prior Mean Value	G-test	df	p-value	G-test (Data)	df (Data)	p-value (Data)
Achievement	1.3645	58.7667%	61.7367%	1.0000	0.0019	7,782.1578	12	0.0000%	7,782.1578	12	0.0000%
Power	1.2011	51.7266%	54.3408%	0.8802	0.0011	6,849.8700	12	0.0000%	6,849.8700	12	0.0000%
Stimulation	0.9574	41.2351%	43.3191%	0.7017	0.0023	5,460.5389	12	0.0000%	5,460.5389	12	0.0000%
Hedonism	0.7495	32.2808%	33.9122%	0.5493	0.0041	4,274.7675	12	0.0000%	4,274.7675	12	0.0000%

Social focused value (self-transcendence)

Performance Indices	
Number of Clusters	5
Mean Purity	92.2402%
Contingency Table Fit	96.1280%
Deviance	565.5160
Hypercube Cells Per State	49.2176

Overall Analysis with [Factor_9]											
Node	Mutual Information	Normalized Mutual Information	Relative Mutual Information	Relative Significance	Prior Mean Value	G-test	df	p-value	G-test (Data)	df (Data)	p-value (Data)
Universalism	1.4537	62.6067%	65.3363%	1.0000	0.0074	8,290.6588	12	0.0000%	8,290.6588	12	0.0000%
Benevolence	1.4005	60.3171%	62.9469%	0.9634	0.0093	7,987.4663	12	0.0000%	7,987.4663	12	0.0000%
Selfdirection	0.9417	40.5563%	42.3245%	0.6478	0.0067	5,370.6438	12	0.0000%	5,370.6438	12	0.0000%
Security	0.7622	32.8244%	34.2555%	0.5243	0.0061	4,346.7565	12	0.0000%	4,346.7565	12	0.0000%

Conservation value

Performance Indices	
Number of Clusters	4
Mean Purity	97.2995%
Contingency Table Fit	97.7822%
Deviance	84.2835
Hypercube Cells Per State	3.9113

Overall Analysis with [Factor_10]											
Node	Mutual Information	Normalized Mutual Information	Relative Mutual Information	Relative Significance	Prior Mean Value	G-test	df	p-value	G-test (Data)	df (Data)	p-value (Data)
Conformity	1.3684	68.4176%	72.8392%	1.0000	0.0057	7,804.0014	9	0.0000%	7,804.0014	9	0.0000%
Tradition	1.1219	56.0940%	59.7192%	0.8199	0.0050	6,398.3259	9	0.0000%	6,398.3259	9	0.0000%

Pleasure seeking

Performance Indices	
Number of Clusters	3
Mean Purity	92.8565%
Contingency Table Fit	66.3534%
Deviance	259.1459
Hypercube Cells Per State	2.6541

Overall Analysis with [Factor_11]

Node	Mutual Information	Normalized Mutual Information	Relative Mutual Information	Relative Significance	Prior Mean Value	G-test	df	p-value	G-test (Data)	df (Data)	p-value (Data)
Q10_enjoy_cafes	0.8659	54.6328%	62.1546%	1.0000	4.2114	4,938.4633	4	0.0000%	4,942.5370	4	0.0000%
Q10_travel_abroad_asap	0.5100	32.1765%	36.6066%	0.5890	3.5241	2,908.5574	6	0.0000%	2,909.1210	6	0.0000%

Risk perception

Performance Indices	
Number of Clusters	5
Mean Purity	90.5524%
Contingency Table Fit	96.4397%
Deviance	267.0344
Hypercube Cells Per State	24.1099

Overall Analysis with [Factor_12]

Overall Analysis with [Factor_12]

Node	Mutual Information	Normalized Mutual Information	Relative Mutual Information	Relative Significance	Prior Mean Value	G-test	df	p-value	G-test (Data)	df (Data)	p-value (Data)
Q8_BecomeIll	1.4852	63.9659%	65.5772%	1.0000	4.1950	8,470.6560	16	0.0000%	8,482.8255	16	0.0000%
Q8_BecomeInfect	1.1817	50.8916%	52.1736%	0.7956	4.0394	6,739.2965	16	0.0000%	6,753.7533	16	0.0000%
Q8_InfectOthers	0.7386	31.8116%	32.6130%	0.4973	5.0201	4,212.6421	16	0.0000%	4,207.7744	16	0.0000%

Appendix 4 - Step 4: Probabilistic Structural Equation Modeling

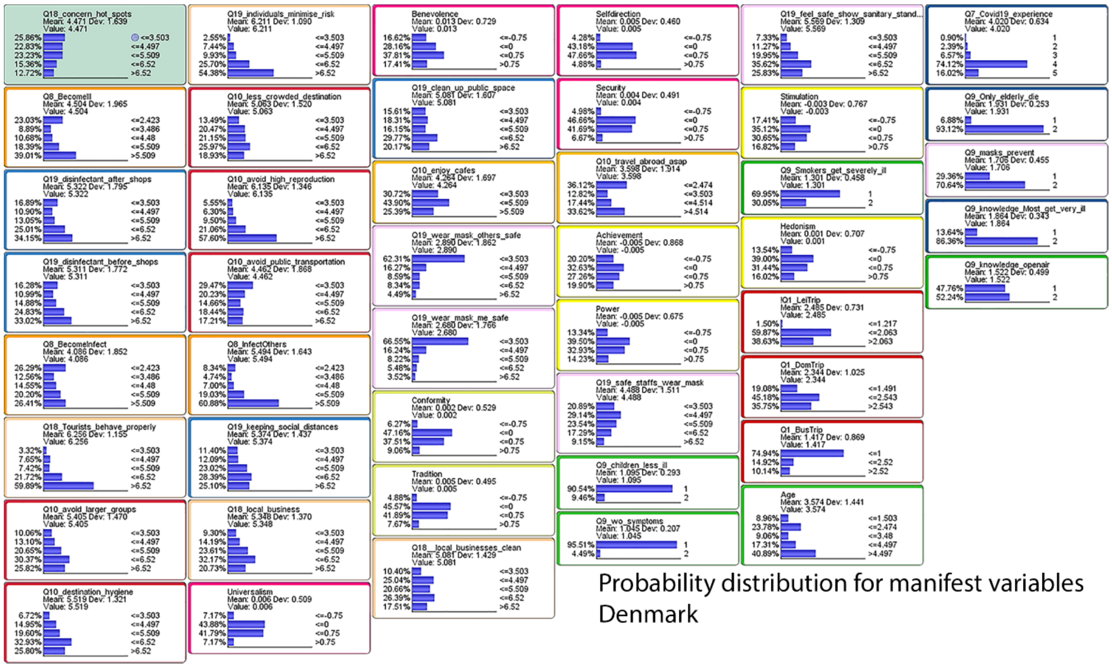

Probability distribution for manifest variables
Denmark

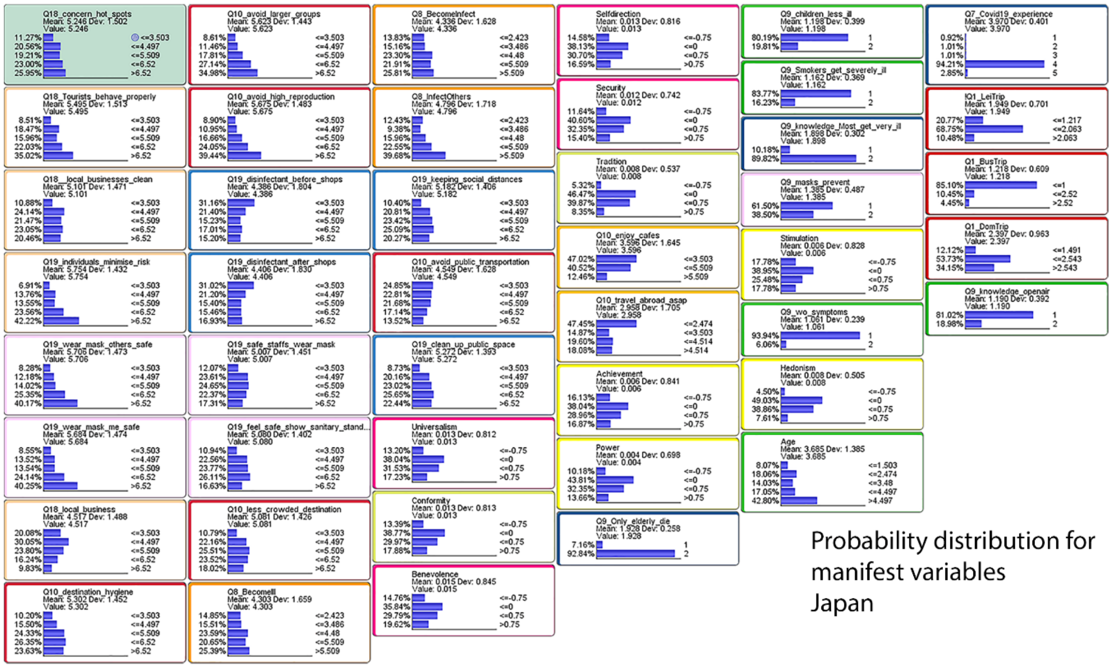

Probability distribution for
manifest variables
Japan

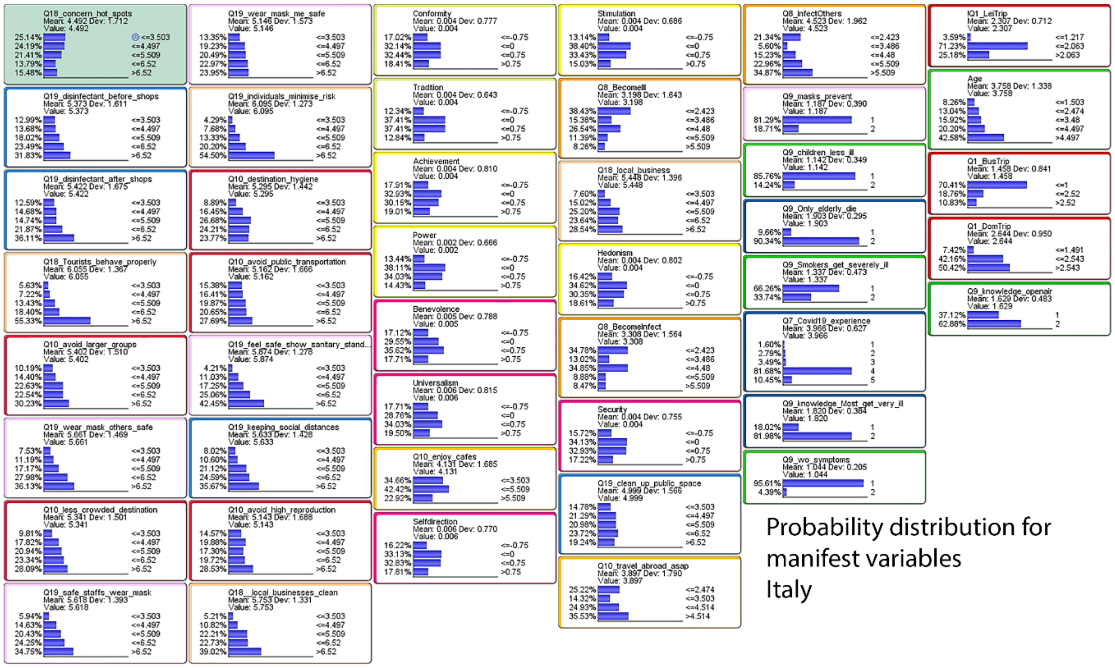

Probability distribution for manifest variables Italy

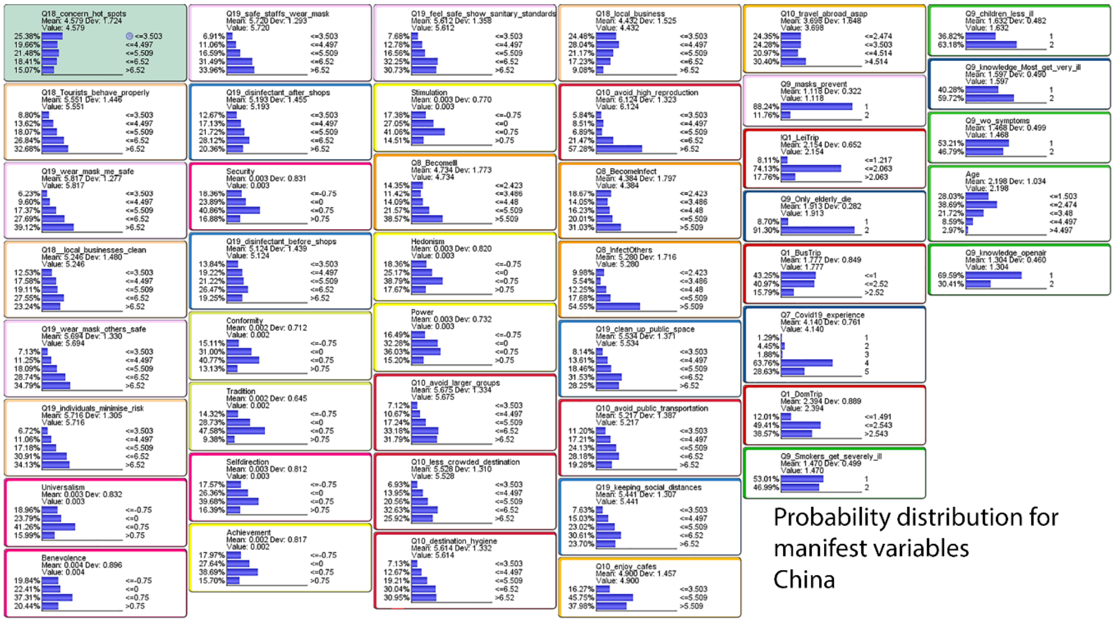

Probability distribution for manifest variables China

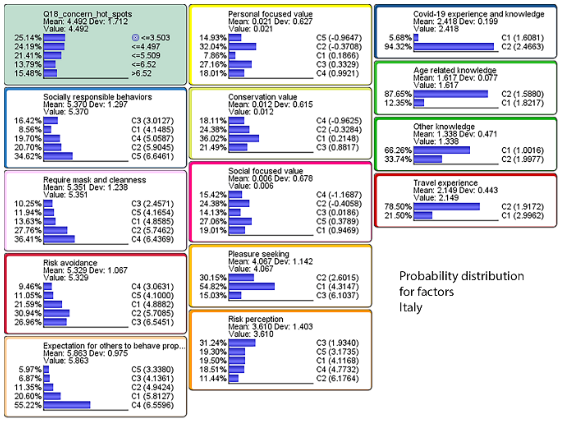

Probability distribution for factors Italy

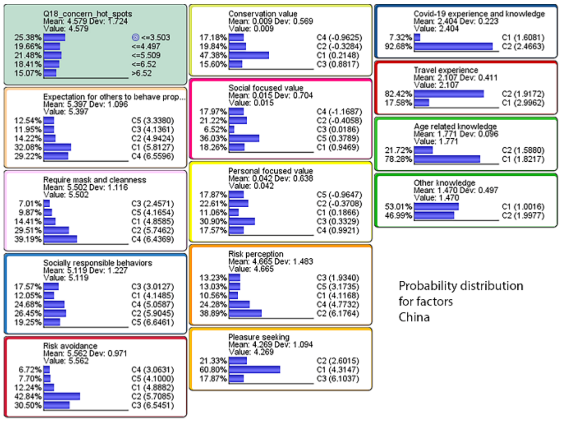

Probability distribution for factors China

Appendix 5 - Step 5: Target mean analysis and total effects

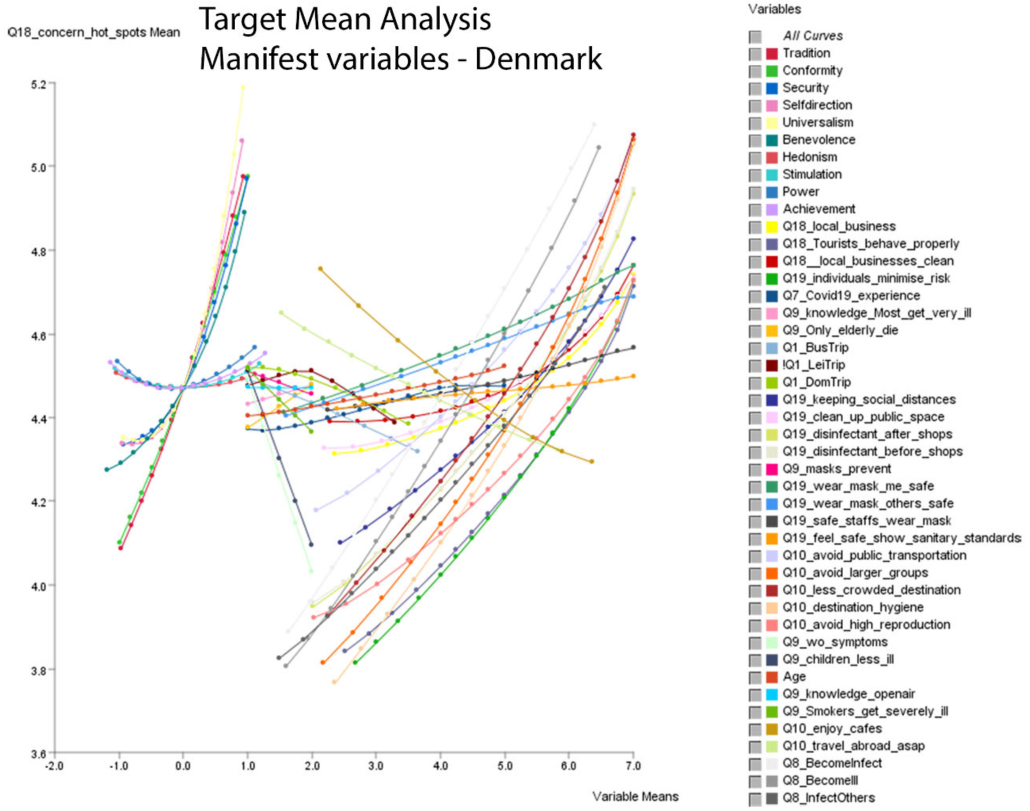

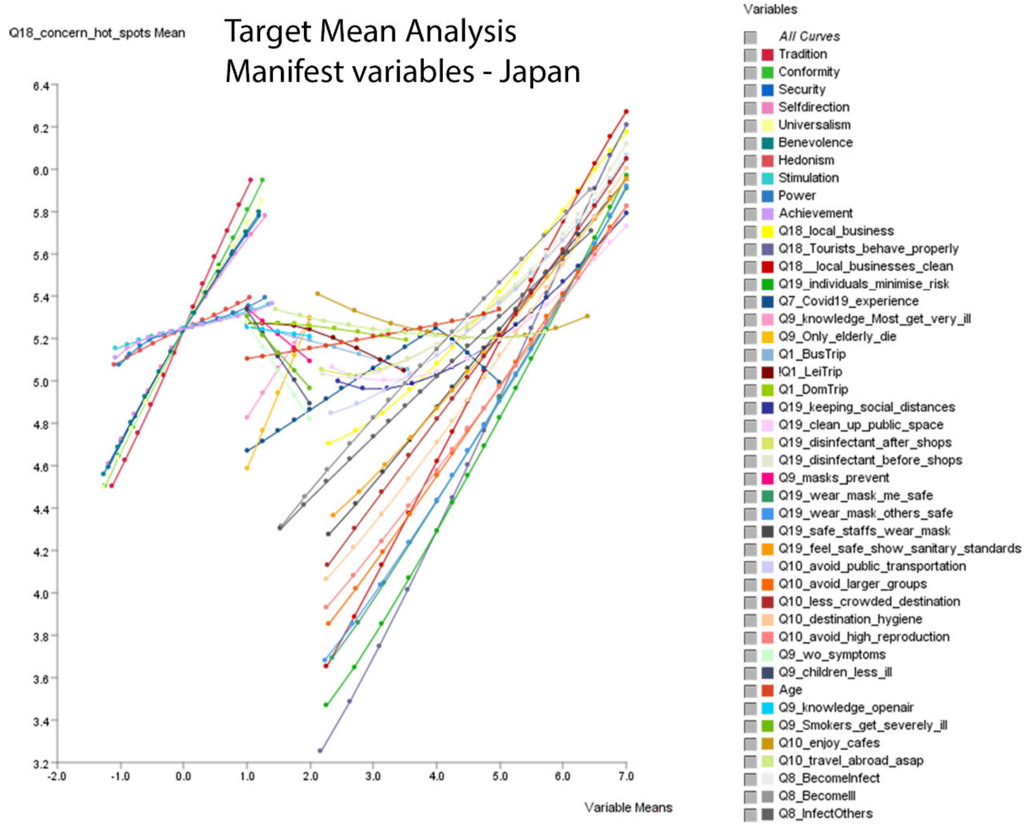

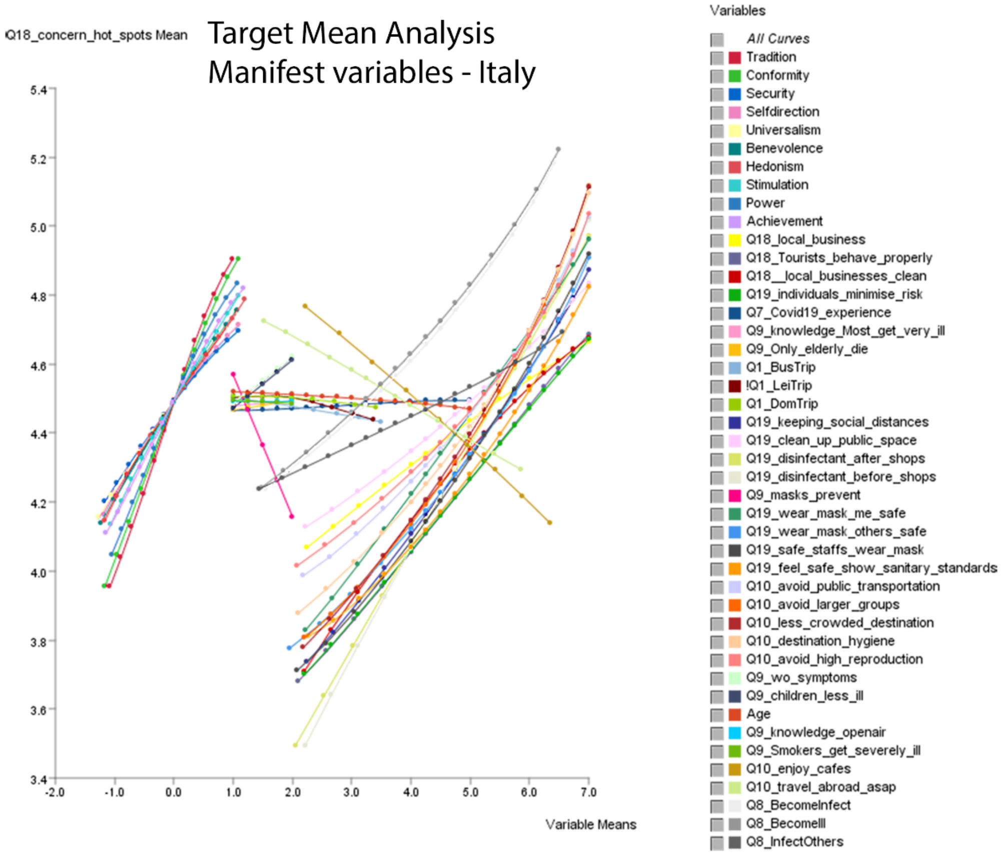
Target Mean Analysis
Manifest variables - Italy
Q18_concern_hot_spots Mean
Variable Means
Variables
All Curves
Tradition
Conformity
Security
Selfdirection
Universalism
Benevolence
Hedonism
Stimulation
Power
Achievement
Q18_local_business
Q18_Tourists_behave_properly
Q18__local_businesses_clean
Q19_individuals_minimise_risk
Q7_Covid19_experience
Q9_knowledge_Most_get_very_ill
Q9_Only_elderly_die
Q1_BusTrip
!Q1_LeiTrip
Q1_DomTrip
Q19_keeping_social_distances
Q19_clean_up_public_space
Q19_disinfectant_after_shops
Q19_disinfectant_before_shops
Q9_masks_prevent
Q19_wear_mask_me_safe
Q19_wear_mask_others_safe
Q19_safe_staffs_wear_mask
Q19_feel_safe_show_sanitary_standards
Q10_avoid_public_transportation
Q10_avoid_larger_groups
Q10_less_crowded_destination
Q10_destination_hygiene
Q10_avoid_high_reproduction
Q9_wo_symptoms
Q9_children_less_ill
Age
Q9_knowledge_openair
Q9_Smokers_get_severely_ill
Q10_enjoy_cafes
Q10_travel_abroad_asap
Q8_BecomeInfect
Q8_BecomeIll
Q8_InfectOthers

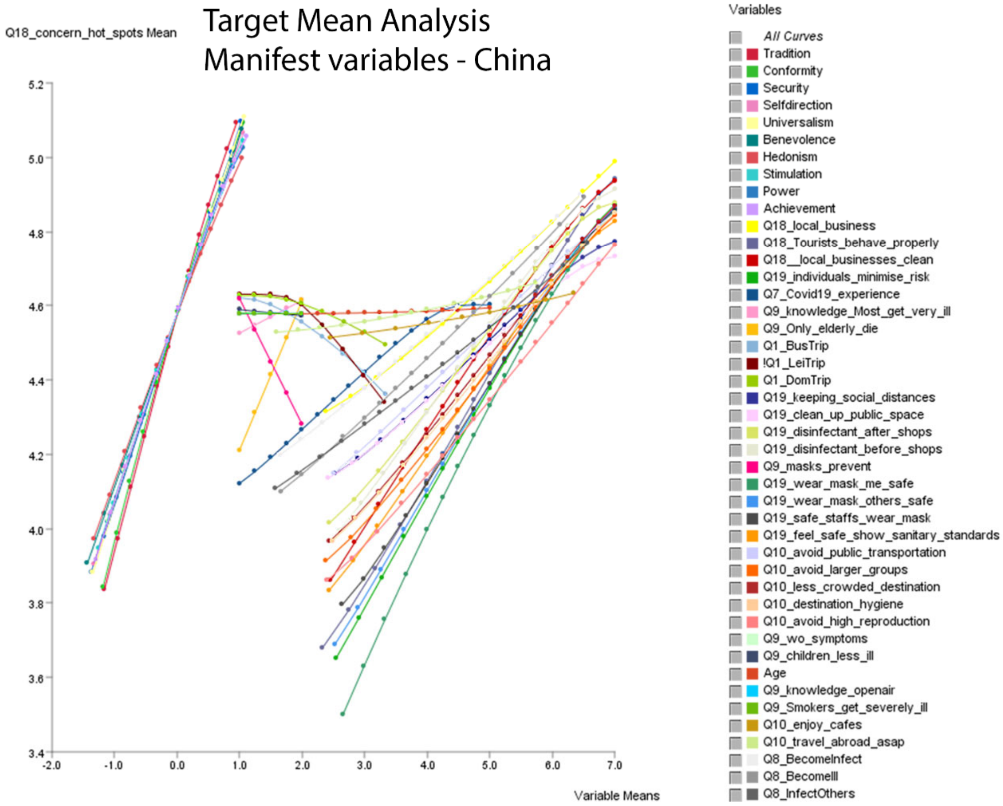
Target Mean Analysis
Manifest variables - China
Q18_concern_hot_spots Mean
Variable Means
Variables
All Curves
Tradition
Conformity
Security
Selfdirection
Universalism
Benevolence
Hedonism
Stimulation
Power
Achievement
Q18_local_business
Q18_Tourists_behave_properly
Q18__local_businesses_clean
Q19_individuals_minimise_risk
Q7_Covid19_experience
Q9_knowledge_Most_get_very_ill
Q9_Only_elderly_die
Q1_BusTrip
!Q1_LeiTrip
Q1_DomTrip
Q19_keeping_social_distances
Q19_clean_up_public_space
Q19_disinfectant_after_shops
Q19_disinfectant_before_shops
Q9_masks_prevent
Q19_wear_mask_me_safe
Q19_wear_mask_others_safe
Q19_safe_staffs_wear_mask
Q19_feel_safe_show_sanitary_standards
Q10_avoid_public_transportation
Q10_avoid_larger_groups
Q10_less_crowded_destination
Q10_destination_hygiene
Q10_avoid_high_reproduction
Q9_wo_symptoms
Q9_children_less_ill
Age
Q9_knowledge_openair
Q9_Smokers_get_severely_ill
Q10_enjoy_cafes
Q10_travel_abroad_asap
Q8_BecomeInfect
Q8_BecomeIll
Q8_InfectOthers
